# Role for the Epidermal Growth Factor Receptor in Chemotherapy-Induced Alopecia

**DOI:** 10.1371/journal.pone.0069368

**Published:** 2013-07-19

**Authors:** Kyle J. Bichsel, Navdeep Gogia, Timothy Malouff, Zachary Pena, Eric Forney, Brianna Hammiller, Patrice Watson, Laura A. Hansen

**Affiliations:** 1 Department of Biomedical Sciences, School of Medicine, Creighton University, Omaha, Nebraska, United States of America; 2 Department of Preventative Medicine and Public Health, School of Medicine, Creighton University, Omaha, Nebraska, United States of America; University of Kentucky, United States of America

## Abstract

Treatment of cancer patients with chemotherapeutics like cyclophosphamide often causes alopecia as a result of premature and aberrant catagen. Because the epidermal growth factor receptor (EGFR) signals anagen hair follicles to enter catagen, we hypothesized that EGFR signaling may be involved in cyclophosphamide-induced alopecia. To test this hypothesis, skin-targeted *Egfr* mutant mice were generated by crossing floxed *Egfr* and *Keratin 14* promoter-driven *Cre recombinase* mice. Cyclophosphamide treatment of control mice resulted in alopecia while *Egfr* mutant skin was resistant to cyclophosphamide-induced alopecia. *Egfr* mutant skin entered catagen normally, as indicated by dermal papilla condensation and decreased follicular proliferation, but did not progress to telogen as did *Egfr* wild type follicles. *Egfr* mutant follicles responded with less proliferation, apoptosis, and fewer p53-positive cells after cyclophosphamide. Treatment of control mice with the EGFR inhibitors erlotinib or gefitinib similarly suppressed alopecia and catagen progression by cyclophosphamide. Secondary analysis of clinical trials utilizing EGFR-targeted therapies and alopecia-inducing chemotherapy also revealed evidence for involvement of EGFR in chemotherapy-induced alopecia. Taken together, our results demonstrated the involvement of EGFR signaling in chemotherapy-induced alopecia, which will help in the design of novel therapeutic regimens to minimize chemotherapy-induced alopecia.

## Introduction

Chemotherapeutic drugs used for cancer treatment often result in side effects due to the death of highly proliferative cells. The nitrogen mustard phosphamide ester, cyclophosphamide, is an alkylating agent that induces apoptosis in rapidly dividing cells, which usually leads to alopecia [1]. Chemotherapy-induced alopecia can cause much anguish among cancer patients; most report at least mild distress [2]. Chemotherapy agents such as cyclophosphamide have high potential to induce chemotherapy-related alopecia [3], [4].

The sensitivity of follicles to chemotherapeutics is believed due to the high proliferation rate in matrix keratinocytes during hair growth. Hair follicles are unique in that they cycle through phases of active proliferation and hair growth (anagen), apoptotic involution (catagen), and rest (telogen) throughout the life of the organism. The hair cycle progresses in synchronous waves early in the lives of mice, becoming less synchronous as the animal ages. Human scalp follicles cycle identically to those in mice, however each follicle cycles independently. During anagen, matrix keratinocytes rapidly divide to produce the growing hair shaft [5]. Anagen matrix keratinocytes are some of the most rapidly dividing cells in the body, with as many as 60% in S-phase [5]. By targeting dividing cells, chemotherapy-induced apoptosis causes aberrant and premature involution of the hair follicle in humans and animal models, although the process occurs more rapidly in the mouse [1], [6], [7]. Despite progress in identifying the histological changes of chemotherapy-induced alopecia, our understanding of its mechanisms has limited the development of strategies to alleviate it.

EGFR signaling is not required for follicular proliferation, although it contributes to follicular differentiation [8]–[10]. In addition, *Egfr* deficient mice exhibit delayed and defective catagen [9], [11]. We hypothesized that EGFR signaling is involved in cyclophosphamide-induced catagen and alopecia. Both skin-targeted *Egfr* mutant mice and EGFR inhibitor-treated mice were resistant to cyclophosphamide-induced alopecia. In order to determine whether these data are relevant clinically, secondary analysis of clinical trials utilizing EGFR-targeting agents together with chemotherapeutics that cause alopecia was undertaken, revealing evidence of a role for EGFR in alopecia in cancer patients.

## Materials and Methods

### Animals

*Egfr* mutant (*Egfr^fl/fl^/Krt14 Cre recombinase ^+^)* and control (*Egfr^wt/wt^/Krt14 Cre recombinase^+^*or *Krt14 Cre recombinase ^-^*) mice on an FVB/N background, or wild type FVB/N mice, were injected intraperitoneally with 150 mg/kg cyclophosphamide (EMD Chemicals, Gibbstown, NJ) or vehicle (water) at P12. Mice were euthanized at various time points after cyclophosphamide treatment. Mice were injected with 250 µg/g body weight of bromodeoxyuridine (BrDU) one hour before euthanasia, and mid-back dorsal skin samples were fixed and sectioned. Three mice per genotype and time point were treated with cyclophosphamide while 2–3 mice per time point and genotype were treated with vehicle alone. For some experiments, the dorsal hair of wild type FVB/N mice was lightly trimmed with electric clippers prior to topical treatment with 100 mg/kg body weight erlotinib (Cayman Chemical Company, Ann Arbor, MI) or 3 mg/kg body weight gefitinib (Tocris Bioscience, Bristol, UK) in 200 µl acetone/DMSO (85∶15), or with the vehicle. Two hours later, the mice were injected with cyclophosphamide. The mice were treated with vehicle or EGFR inhibitors daily until euthanasia at P15 or P21. Dorsal hair was lightly trimmed as needed prior to each of the topical treatments. Mice were photographed at euthanasia, dorsal skin sections were prepared for hematoxylin and eosin staining and for immunofluorescence, and skin was removed for immunoblotting.

### Ethics Statement

The research did not involve human subjects. All animal work was approved by Creighton University’s Institutional Animal Care and Use Committee (protocols 0809.2 and 0690.2).

### Histological Examination and Histomorphometry

Hair cycle staging, morphological analyses, and histomorphometry were performed using previously described morphological markers [12], [13] using hematoxylin and eosin stained sections (American Histolabs, Gaithersburg, MD. Hair follicle dystrophy scores were calculated by assigning numerical values to specific hair cycle stages as described previously [13]. Hair follicles were scored as follows: dystrophic anagen = 1–3, dystrophic catagen = 4–6, dystrophic telogen = 7 and new anagen = 8. The mean of scored hair follicles for a group was used as the dystrophy score.

### Endogenous Alkaline Phosphatase Activity

Alkaline phosphatase activity was assessed in cryosections following incubation with 5-bromo-4-chloro-3-indolyl phosphate/p-nitroblue tetrazolium chloride (Sigma, St. Louis, MO), as described in [14].

### TUNEL Assays

Apoptotic cells were identified using DeadEnd Fluorometric TUNEL (Promega, Madison, WI). Quantification of TUNEL-positive cells was performed on at least 7 hair follicles per mouse (N = 3 mice). Matrix keratinocytes, where morphologic characteristics of the bulb were clear, were counted in the proximal hair follicle using DAPI and quantification of TUNEL^+^ nuclei.

### Immunofluorescence

Localization of p53 protein was assessed in cryosections or in formalin-fixed paraffin-embedded sections using anti-murine p53 (Santa Cruz, Santa Cruz, CA) and Tyramide Signal Amplification (Promega, Madison, WI) as in [15], [16]. BrDU (BD Bioscience, San Jose, CA) and PCNA (proliferating cell nuclear antigen)(Santa Cruz Biotechnologies, Santa Cruz, CA) immunofluorescence was performed using standard techniques in paraffin-embedded sections.

### Immunoblotting

Immunoblotting for phospho-EGFR on Tyr-992 (Cell Signaling, Beverly, MA) was performed using standard techniques. The evenness of loading and transfer was assessed using Ponceau S (Sigma Chemical Company, Saint Louis, MO) staining.

### Clinical Trial Analysis

Electronic databases were used to identify published human studies incorporating anti-EGFR therapy combined with alopecia-inducing chemotherapy (complete search terms in Table S1). The search was restricted to studies in English. The National Institutes of Health Clinical Trials (www.clincialtrials.gov)(January 2000 to March 2012) and MEDLINE (January 1946 to March 2012) databases were searched. Inclusion criteria (established by authors K.J.B. and Z.P.) included randomized patient assignment to treatment arms, trials that included a matched control arm lacking EGFR-targeted therapy, and anti-EGFR therapy administered concurrently with or prior to chemotherapy. Studies included in the final analyses contained complete descriptions of experimental design and reporting of adverse events. Exclusion criteria were established (by authors K.J.B. and Z.P.) to eliminate trials that did not include adequate controls, did not combine anti-EGFR treatments with alopecia-inducing chemotherapy or used non-specific inhibitors of EGFR, among other criteria described in Table S2. The reference list of all excluded studies with explanations is included in Table S4. Studies were not excluded based on sex or age of the participants. The authors recognize limitations in the analysis including inconsistencies in alopecia reporting across all trials and the lack of standardized alopecia severity criteria. Studies were not excluded due to these limitations. Two by Two contingency tables were generated (P.W. and K.J.B.) from the adverse events tables for each trial. Chi-square testing with Yates’ correction was performed for significance.

## Results

### EGFR was Required for Alopecia and Progression through Catagen after Cyclophosphamide Administration

**Figure 1:**
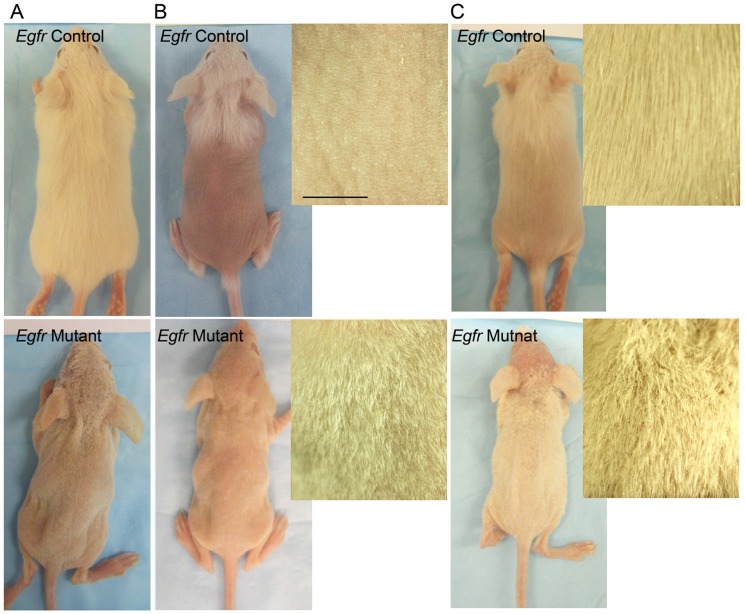
*Egfr* deficient hair follicles were resistant to cyclophosphamide-induced alopecia. *Egfr* mutant (bottom) and control mice (top) were injected with cyclophosphamide (B,C) or vehicle (A) alone and photographed 8d (A–B) or 15d (C) later. Insets, magnification of dorsal skin. Scale bar indicates 5 mm.

To investigate the involvement of EGFR in cyclophosphamide-induced alopecia, mice with skin-targeted deletion of *Egfr* were generated by crossing *Krt14-Cre Recombinase* transgenics [17] with a line where loxP sites flank exon 3 of the *Egfr* [18]. The cross generated a frame shift mutation, resulting in a nonfunctional receptor. EGFR levels were 90% reduced in *Egfr^fl/fl^/Cre^+^* mutant epidermis, as shown by densitometry of multiple immunoblots (Figure S1) [19]. *Egfr* deficient mice displayed a fragile hair phenotype characterized by short, wavy hairs covering the body and curly vibrissae (Figure 1A bottom), similar to other models of EGFR deficiency [8], [11], [20].

*Egfr* mutant and control mice were injected with vehicle or with cyclophosphamide at 12 days of age (P12), prior to onset of any hair cycle abnormalities (unpublished data). *Egfr* control mice exhibited hair loss beginning 5–6 days after cyclophosphamide (data not shown), with complete alopecia over the dorsal region between the neck and just proximal to the tail by 8 days (Figure 1B top). In contrast, *Egfr* mutant mice retained their short, wavy coat at 8 days (Figure 1B bottom) and throughout the 15 days observation period (Figure 1C bottom, Figure S2). Hair began regrowing in the cyclophosphamide treated *Egfr* controls by 15 days (Figure 1C top).

**Figure 2:**
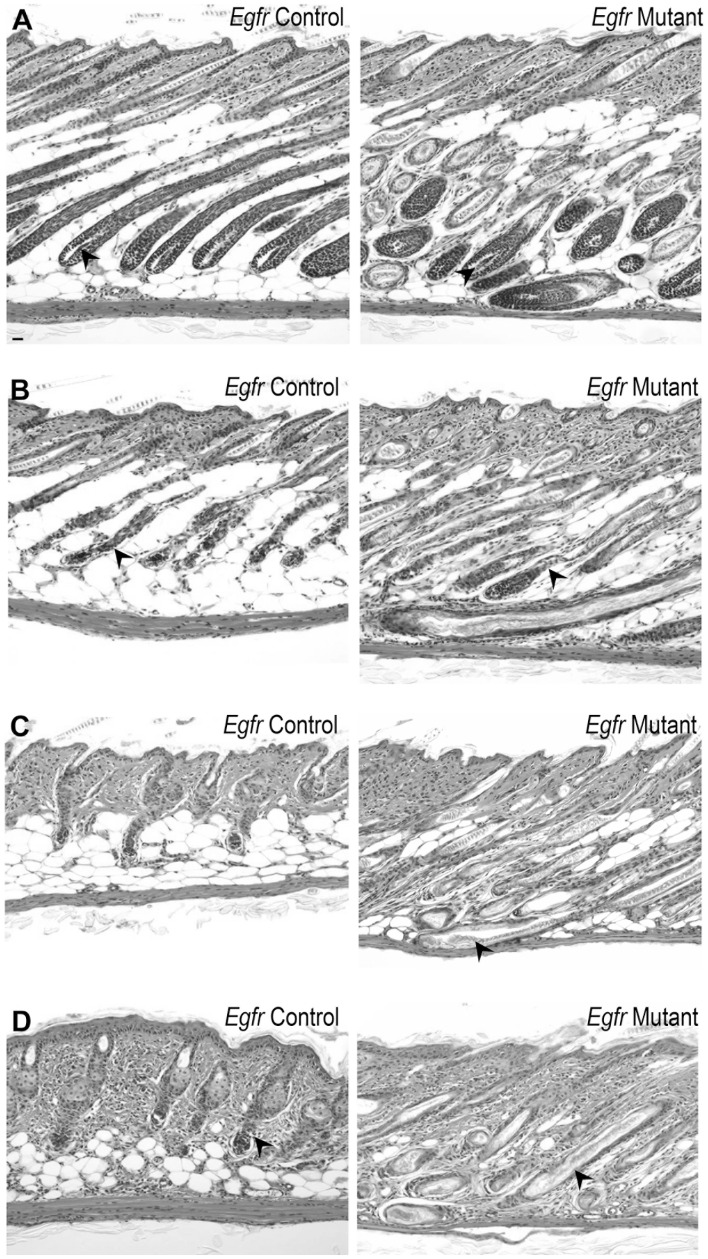
*Egfr* mutant follicles did not progress through catagen following cyclophosphamide. Hematoxylin and eosin stained sections from mutant (panels on right) and control (panels on left) mice after cyclophosphamide (B-D) or vehicle (A). Vehicle mice displayed anagen 2d post-injection (A, arrowheads). *Egfr* controls (B left) exhibited follicular distortion (arrowhead) and regression, while mutants (B right) had follicular distortion (arrowhead) at 2 d after cyclophosphamide. By 4d, controls (C left) displayed late dystrophic catagen, while mutants (C right) maintained elongated follicles (arrowhead). Controls initiated secondary recovery with anagen by 8 d (D left), indicated by the bulb position at dermis and subcutis border (arrowhead). Mutant follicles retained hair at 8 d (D right, arrowhead). Scale bar indicates 100 µm.

Histological examination revealed fully elongated follicles with the spindle shaped dermal papilla fully enclosed by matrix keratinocytes in vehicle treated control and mutant mice at 2 days after injection (Figure 2A, arrowheads), indicating normal anagen VI [12], [13]. Consistent with previous reports of *Egfr* null mice [8], [9], [11], [21], *Egfr* mutant skin displayed follicular disorganization with erratic follicle placement (Figure 2A, right panel). Two days following cyclophosphamide, a majority of *Egfr* control hair follicles were in mid-dystrophic catagen with the follicular distortion (Figure 2B left panel, arrowhead) and marked follicular regression (Figure 2B left panel, note hair follicle length compared to Figure 2A left panel) characteristic of cyclophosphamide side effect [6], [13]. Late-dystrophic catagen occurred in *Egfr* control skin by 4 days after cyclophosphamide with regression nearly completed (Figure 2C left panel). By 8 days after cyclophosphamide, secondary recovery of a new anagen began, indicated by proliferation-dependent elongation between club hair and dermal papilla, and the bulb positioning at the border between dermis and subcutis (Figure 2D left panel, arrowhead). *Egfr* mutant hair follicles failed to complete catagen. At 2 d after cyclophosphamide, mutants sustained subtle changes associated with catagen initiation with some follicular distortion (Figure 2B right panel, arrowhead). However, mutant hair follicles remained elongated. By 4 days after cyclophosphamide, *Egfr* mutant follicles remained in the deep subcutis (Figure 2C right, arrowhead) and did not progress into later stages of catagen. Eight days following cyclophosphamide, mutant hair shafts remained in the follicle (Figure 2D right, arrowhead). No characteristics of progression to late catagen were observed, with hair follicles extended into the subcutis and decreasing cellularity in the proximal hair follicle (Figure 2D right). At 15 days after cyclophosphamide, controls progressed to late anagen with formation of mature hair follicles and production of new hair shafts, while mutants retained their hair shafts and lacked characteristics of anagen reinitiation (Figure S2A–B).

**Figure 3:**
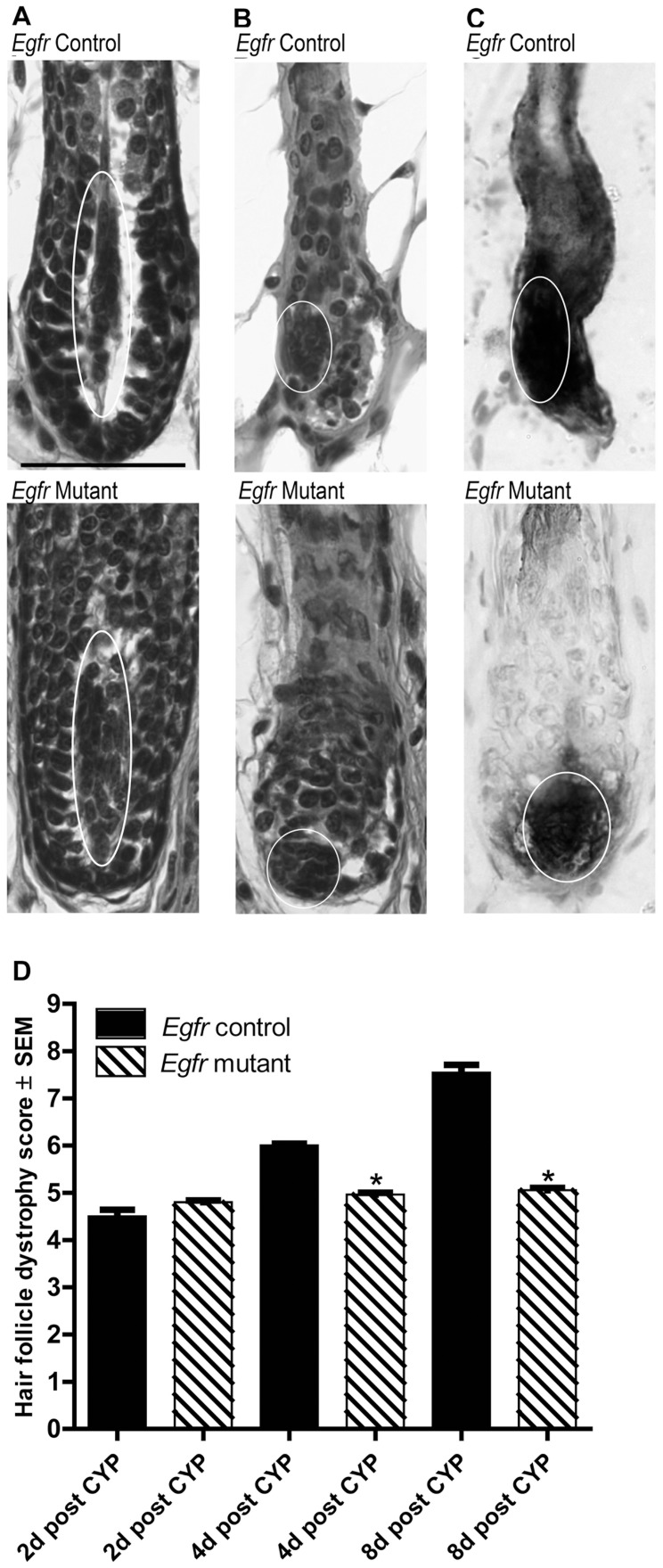
Cyclophosphamide-treated *Egfr* mutants displayed characteristics of early catagen with dermal papilla condensation 2d post administration. A–B) Hematoxylin and eosin stained sections of vehicle treated controls (A top) and mutants (A bottom) displayed anagen dermal papilla with spindle shapes (ovals) surrounded by matrix keratinocytes. Control (B top) and mutant (B bottom) dermal papilla condensed to the proximal hair follicle by 2d following cyclophosphamide (ovals). Endogenous alkaline phosphatase confirmed dermal papilla position in controls (C top, oval) and mutants (C bottom, oval) at 2d. Scale bar indicates 50 µm. D) Hair follicle dystrophy score calculated following cyclophosphamide (CYP) as follows: dystrophic anagen = 1–3, dystrophic catagen = 4–6, dystrophic telogen = 7 and new anagen = 8. *Significant using Student’s t-test, where P≤0.05.

Additional assessments of dermal papilla positioning after cyclophosphamide were undertaken to further characterize the hair cycle stage. As described previously [12], [13], the dermal papillae of control hair follicles transitioned from an elongated spindle shape surrounded by matrix keratinocytes to a more ball or onion-like shape as the hair follicles moved from anagen to early- and mid-catagen in response to cyclophosphamide (Figure 3A,B top panels, ovals). *Egfr* mutants similarly transitioned to a condensed dermal papilla (Figure 3A,B bottom panels, ovals). Analysis of endogenous alkaline phosphatase histochemistry confirmed the position and shape changes of the dermal papillae in *Egfr* controls (Figure 3C top, oval) and mutants (Figure 3C bottom, oval) after cyclophosphamide.

Quantitative histomorphometry (Figure S3) and calculation of a hair follicle dystrophy score (Figure 3D) confirmed our qualitative observations of hair cycle stage. Two days after cyclophosphamide, most mutant and control follicles were similarly in mid-dystrophic catagen, indicated by a hair follicle dystrophy score of 4–6 (Figure 3D). The hair follicle dystrophy score revealed progression of control follicles through catagen, with secondary recovery by 8 days (Figure 3D and Figure S3). However, mutant follicles maintained a hair follicle dystrophy score of approximately 5 for the duration of the experiment, indicating dystrophic catagen (Figure 3D). Vehicle-treated mutant and control hair follicles at 2 days were similarly in late anagen (Figure S3).

### Proliferation in *Egfr* mutant follicles was decreased following cyclophosphamide treatment

**Figure 4:**
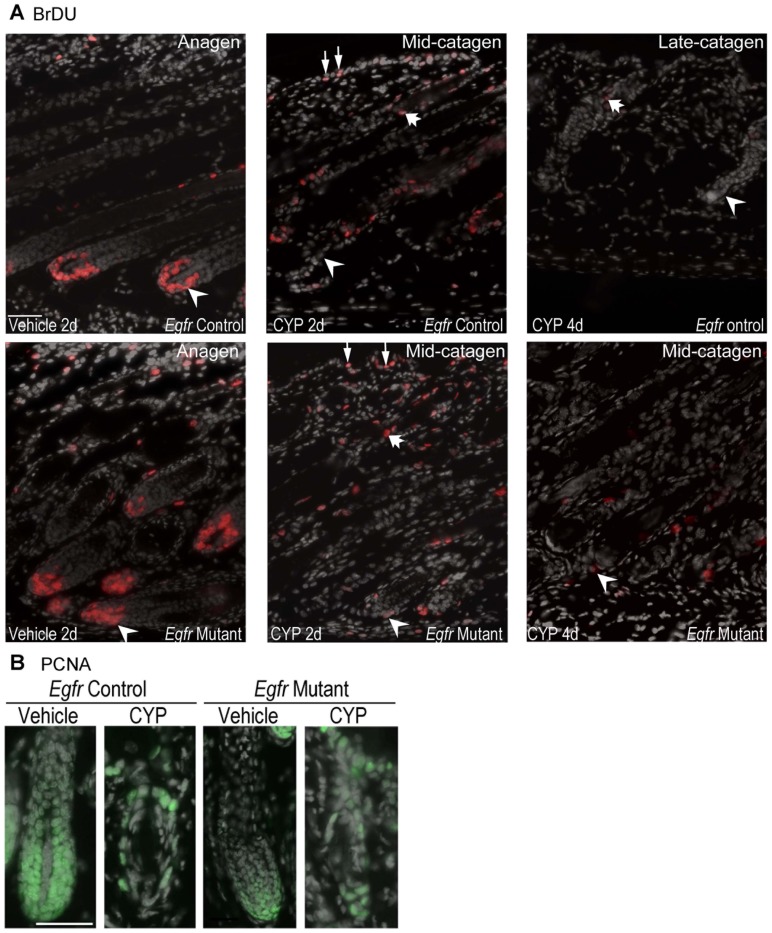
Proliferation in *Egfr* controls and mutants was decreased following cyclophosphamide. BrDU (A) and PCNA (B) immunofluorescence was performed in vehicle and cyclophosphamide treated control and mutant mice euthanized at 2 (A-B) or 4 d (A). A) Arrowheads indicate BrDU-labeled matrix keratinocytes, thin arrows BrDU positive epidermal keratinocytes and fat arrows BrDU positive keratinocytes in the distal follicle. BrDU positive cells are red with nuclear DAPI gray. B) PCNA labeled nuclei are green with nuclear DAPI in gray. CYP indicates cyclophosphamide. Scale bar indicates 50 µm.

To determine whether EGFR deficiency attenuated cyclophosphamide-induced alopecia by altering cell cycle, we examined proliferation following cyclophosphamide treatment. Analysis of BrDU incorporation revealed many vehicle-treated mutant and control matrix cells in S-phase 2 days after treatment (Figure 4A, see arrowheads in panels on left), indicating a typical anagen VI [5]. Following cyclophosphamide, the number of BrDU positive keratinocytes in the hair bulb was strikingly reduced in both controls and mutants (Figure 4A middle panels, arrowheads). However, BrDU positive cells remained in the distal hair follicle and epidermis of both genotypes (Figure 4A middle panels, fat arrow and thin arrows, respectively). Progression to late stages of catagen by 4 days after cyclophosphamide treatment in the control skin was associated with the near cessation of proliferation in the hair follicle (Figure 4A top right, thick arrow). Follicular proliferation resumed at initiation of secondary recovery and new anagen at 8 days (data not shown). Mutant follicles remaining in mid-catagen retained a small number of BrDU positive cells 4 days following cyclophosphamide (Figure 4A bottom right, arrowhead), fewer than at 2 days. The decrease in the proliferation index at 2 days is consistent with catagen entry in cyclophosphamide treated mutants.

Another proliferation marker, PCNA, was also used to identify cycling cells in vehicle and cyclophosphamide follicles. PCNA labeling was decreased in mutant compared to control bulbs after vehicle treatment (Figure 4B). In contrast to BrDU labeling, cyclophosphamide treatment reduced PCNA labeling in control but not mutant follicles (Figure 4B). Thus, decreased follicular proliferation may be a factor in the resistance of the *Egfr* mutants to cyclophosphamide-induced alopecia.

### *Egfr* Deficient Follicles were Resistant to Cyclophosphamide-induced Follicular Apoptosis

**Figure 5:**
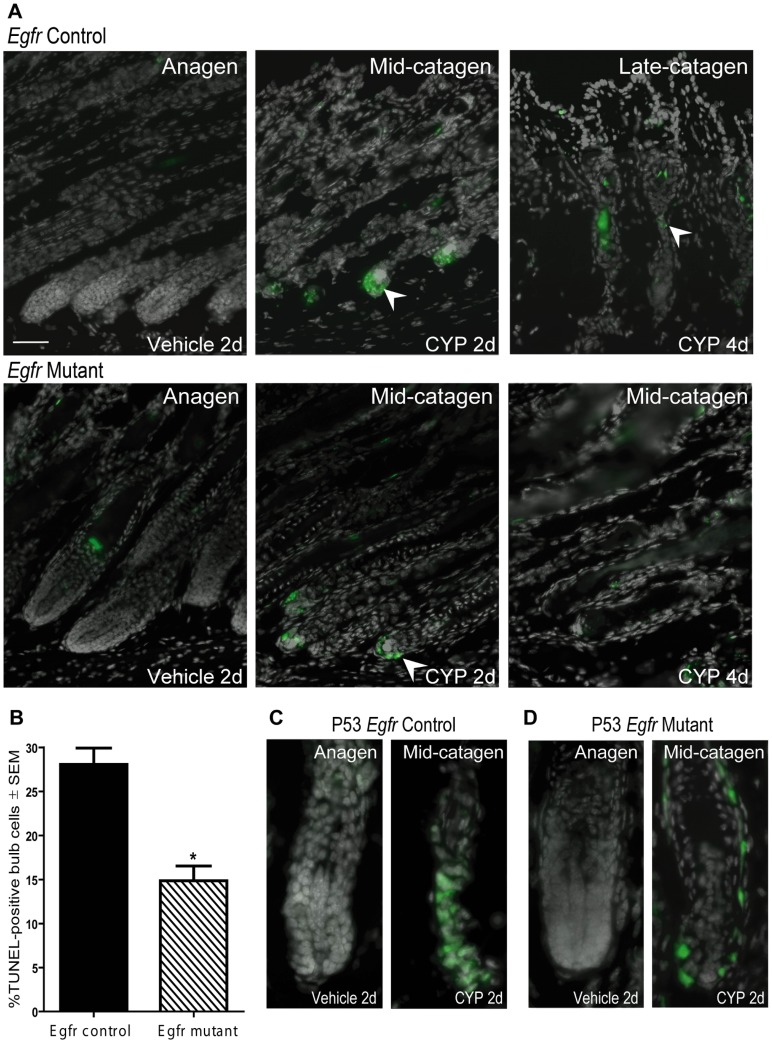
Genetic deletion of *Egfr* suppressed cyclophosphamide-induced follicular apoptosis and p53 immunopositivity. Control (A top panels, C) or mutant (A bottom panels, D) skin, 2 (A left and middle panels,B-D) or 4d (A right panels) after vehicle or cyclophosphamide labeled for TUNEL in green with DAPI in gray (A) or p53 in green with DAPI in gray (C-D). TUNEL-positive cells 2 d after cyclophosphamide (arrowheads), were reduced at 4d (A). B) Quantification of TUNEL-positive bulb cells at 2d (N = 3 mice). *Significantly different using Student’s t-test, where P≤0.05. p53 localized to the bulb of controls (C right) and mutants (D right) 2d following cyclophosphamide (arrowheads). CYP indicates cyclophosphamide treatment. Scale bar indicates 50 µm.

Completion of catagen requires apoptosis of the lower portion of the follicle to allow involution. The vehicle-treated mice of both genotypes at 2 days post-cyclophosphamide injection did not show detectable apoptotic cells by TUNEL assay (Figure 5A left panels). Cyclophosphamide treatment increased apoptosis in the matrix of both genotypes that peaked at 2 days (Figure 5A middle panels, arrowheads). Quantification of bulb apoptotic cells at 2 days after cyclophosphamide revealed twice as much apoptosis in controls compared to the mutants. Approximately 28% of EGFR proficient follicular matrix cells were TUNEL positive at this time point while only 14% TUNEL-positive cells were detected in the mutants (Figure 5A–B). Catagen progression in controls was accompanied by fewer TUNEL-positive cells at day 4 compared to 2 days (Figure 5A top right arrowhead compared to top middle panel). The number of TUNEL-positive cells declined after 2 d in mutant follicles without progression to telogen or follicle shortening (Figure 5A and Figure 2C,D right panels). Thus, loss of EGFR suppressed cyclophosphamide-induced apoptosis and caused resistance to alopecia, suggesting EGFR plays a pro-apoptotic role in chemotherapy-induced alopecia.

### Fewer p53-positive Cells were Detected in Cyclophosphamide-treated *Egfr* Mutants Compared to *Egfr* Controls

p53 stabilization following cyclophosphamide-induced DNA damage triggers apoptosis [22]. p53-deficient mice are resistant to chemotherapy-induced apoptosis and alopecia [4], [15]. We hypothesized that EGFR may be involved in p53 up-regulation after cyclophosphamide. Vehicle-treated anagen follicles of both genotypes exhibited little p53 immunopositivity (Figure 5C–D and Figure S4A–B, fewer than one p53-positive cell/bulb). Two days following cyclophosphamide, many p53-positive cells were localized to control follicles (Figure 5C right) while mutants displayed fewer p53-positive cells (Figure 5D right). p53 was localized to the outer root sheath of mutants with fewer p53-positive cells in the lower follicle in mutants compared to controls (Figure 5C,D). p53-positivity declined in both genotypes at later times (data not shown). Thus, genetic deletion of *Egfr* led to resistance to alopecia and less p53-positivity. *Fas*, *FasL* and *Bax* transcripts were measured by real-time RT-PCR at 2 days following cyclophosphamide to determine the possible mechanism for apoptosis in follicular keratinocytes. No significant differences were observed between the genotypes (not shown).

### EGFR Targeted Therapy Influences Alopecia Rates

To determine whether EGFR-targeted therapy reduces alopecia in cancer patients treated with chemotherapy, secondary analysis of clinical trials was conducted according to the recommendations in Stroup et. al. [23]. NIH Clinical Trials and MEDLINE databases were searched for trials utilizing an EGFR-targeting agent and alopecia-inducing chemotherapeutic drug as described in Table S1. Table S2 describes the criteria for further analysis, including concurrent or prior treatment with EGFR-targeting inhibitor or neutralizing antibody, randomization of patients, and use of matched control groups. After applying the inclusion and exclusion criteria (Table S2 and Methods), the searches yielded 9 studies, 3 with small molecule inhibitors and 6 with neutralizing antibodies.

A trend of reduced alopecia with EGFR targeting appeared in the trials utilizing small molecule inhibitors of EGFR. Among these, daily administration of gefitinib at 500 mg to several hundred patients with non-small-cell lung cancer resulted in a significant decrease in alopecia in patients (P = 0.032 using a Chi-square test with Yates correction). Only 1.5% of patients given 500 mg gefitinib developed alopecia, while 4.7% of patients without the inhibitor had hair loss (Table 1) [24]. The two other trials using daily administration of gefitinib or erlotinib had similar, but nonsignificant, trends of decreased alopecia with inhibition of EGFR. In a phase II trial for breast cancer patients administered gefitinib, an alopecia rate of 40% was compared to 52% without gefitinib (Table 1) [25]. Administration of erlotinib, carboplatin, and paclitaxel to patients with advanced non-small-cell lung cancer revealed 49.8% of patients suffered alopecia versus 56.7% of patients not administered erlotinib (Table 1) [26].

**Table 1:** EGFR-targeted therapy and alopecia rates during chemotherapy.

Study Population	EGFR-targetedtherapy	Additional chemotherapeutic agents^d^		% of patients with alopecia (N)		P-value
				EGFR- targeted therapy	Control	
EGFR specific tyrosinekinase inhibitors						
Herbst et al. 2004 [24]	Gefitinib^a^	Carboplatin^1^, Paclitaxel^2^		500 mg Gefitinib: 1.5% (347) 250 mg Gefitinib: 4.7% (345)	Placebo: 4.7% (345)	500 mg Gefitinib: 0.032*250 mg Gefitinib: 1.0
Guarneri et al. 2008 [25]	Gefitinib^a^	Epirubicin^3^, Paclitaxel^2^		41.0% (32)	52.0% (31)	0.534
Herbst et al. 2005 [26]	Erlotinib^a^	Carboplatin^1^, Paclitaxel^2^		49.8% (209)	56.7% (208)	0.184
EGFR neutralizing antibodies						
NCT00154102, Van Cutsem et al. 2009 [27]	Cetuximab^b^	Irinotecan^4^, 5-Fluorouracil^5^, Leucovorin	37.3% (599)		38.0% (599)	0.905
NCT00042939	Cetuximab^b^	Irinotecan^4^, Docetaxel^2^	48.9% (46)		54.4% (48)	0.843
NCT00148798, Pirker et al. 2009 [28]	Cetuximab^b^	Cisplatin^1^, Vinorelbine^6^	19.5% (557)		19.0% (568)	0.873
NCT00122460, Vermorken et al. 2008 [29]	Cetuximab^b^	Cisplatin^1^, Carboplatin^1^, 5-Fluorouracil^5^	12.3% (222)		7.0% (220)	0.079
NCT00115765, Hecht et al. 2009 [30]	Panitumumab^c^	Bi-monthly Irinotecan^4^+5-Fluorouracil^5^+ Leucovorin or Oxaliplatin^1^+5-Fluorouracil^5^+ Leucovorin, All patients received Bevacizumab^7^	21.4% (518)		16.9% (510)	0.075
Okines et al. 2010 [31]	Panitumumab^d^	Epirubicin^3^, Oxaliplatin^1^, Capecitabine^5^ (Dose de-escalation groups used)	94.0% (16)		85.0% (13)	0.849

a Daily,

b Weekly,

c Bi-monthly,

d Every 3 weeks, unless noted.

1 Alkylating agent,

2 Taxane,

3 Anthracycline,

4 Topoisomerase inhibitor,

5 Nucleoside analog,

6 Vinca alkyloid,

7 Anti-angiogenic antibody.

* Significantly different by Chi-square test with Yates’ correction P≤0.05.

Neutralizing antibodies against EGFR, however, showed no consistent protection from chemotherapy-induced alopecia. Cetuximab administered along with the topoisomerase inhibitor irinotecan, the nucleoside analog 5-fluorouracil, and leucovorin in a large trial of patients with metastatic colorectal cancer resulted in alopecia in 37.3% of the patients compared to 38.0% without cetuximab [27]. A smaller trial of patients with metastatic pancreatic cancer administered cetuximab, irinotecan and docetaxel a taxane, also had lower alopecia rates 48.9% with cetuximab compared to 54.4% without cetuximab (Table 1), although the difference was not significant. The other four trials utilizing cetuximab or panitumumab reported nonsignificantly higher rates of alopecia upon EGFR targeting (Table 1) [24]–[31]. One of these trials (NCT00115765) incorporated the largest numbers of patients, more than 500 per treatment arm, and had a P value approaching significance at P = 0.075 by Chi square test, and a 4.5% higher rate of alopecia with panitumumab.

### EGFR Inhibition Reduced Cyclophosphamide-induced Alopecia in the Mouse

**Figure 6:**
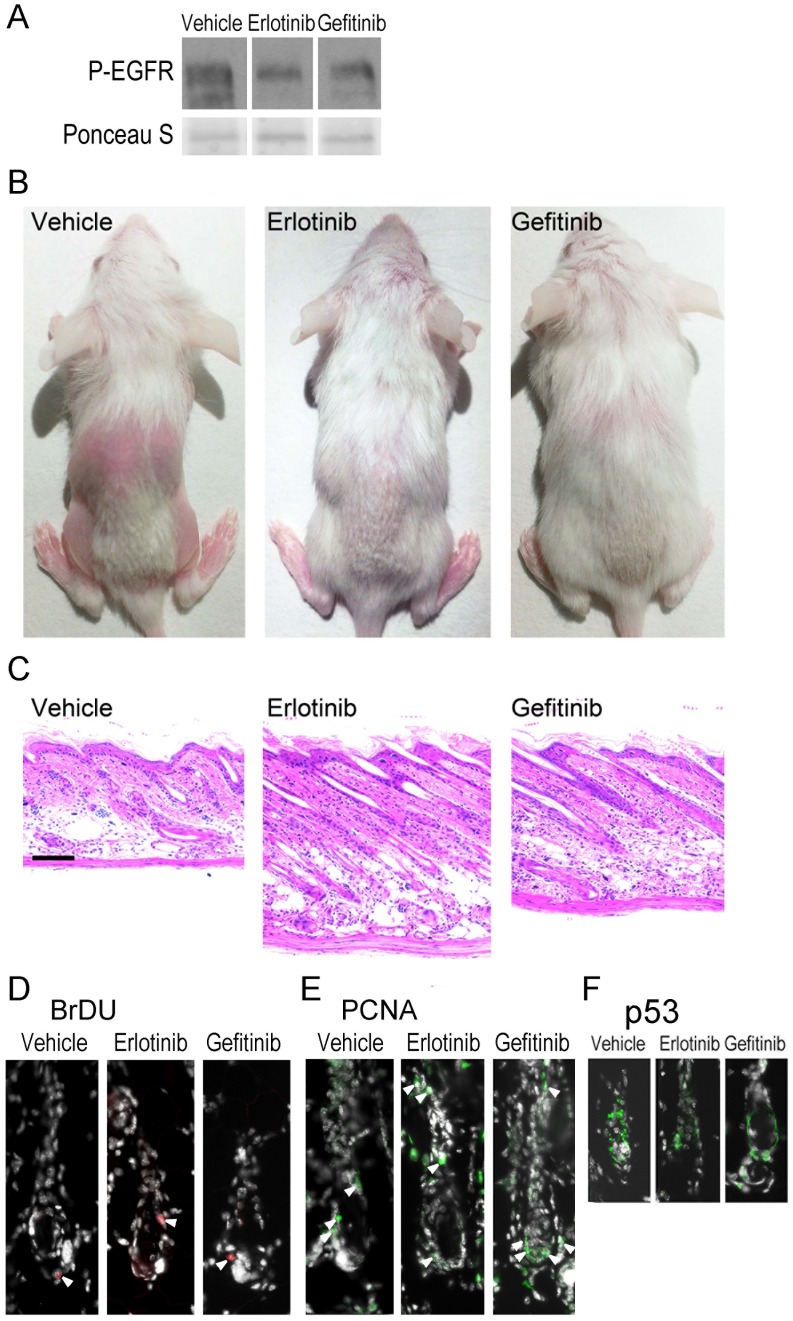
Inhibition of EGFR reduces cyclophosphamide-induced alopecia. Beginning at P12, groups of FVB/N mice were topically treated each day on the dorsal skin with gefitinib, erlotinib, or with the vehicle alone (the dorsal hair was lightly trimmed with electric clippers prior to the first treatment). Two hours after the first treatment, the mice were injected with cyclophosphamide. The mice were monitored daily for alopecia, and euthanized 3 (A,D-F) or 9 d (B-C) later. The mice were photographed (B), skin removed for immunoblotting with the indicated antibodies (A), dorsal skin sections stained with hematoxylin and eosin (C), or immunofluorescence for BrDU (D), PCNA (E), or p53 (F) performed. Arrowheads (D-E) indicate proliferating cells. Images are representative of the two-three mice per group. Scale bar indicates 100 µm.

In order to determine whether EGFR inhibitors could block cyclophosphamide-induced alopecia, groups of wild type mice were topically treated with EGFR inhibitors erlotinib, gefitinib or vehicle alone prior to cyclophosphamide treatment, and alopecia monitored. Mice were treated daily with the inhibitors throughout the course of the experiment, resulting in decreased phospho-EGFR on immunoblot, used as a measure of EGFR activity, when compared to the vehicle treated mice (Figure 6A). After 3d of either erlotinib or gefitinib treatment, phospho-EGFR was reduced by approximately 60% when compared to the vehicle treated control (Figure 6A).While cyclophosphamide-treated mice began to lose hair by 6 d after treatment regardless of EGFR inhibitor status (not shown), alopecia was more pronounced in the vehicle-treated compared to the inhibitor-treated mice. By 9 d after cyclophosphamide injection, hair loss was substantial in the vehicle treated and cyclophosphamide injected mice (Figure 6B left). In contrast, EGFR inhibitor treated and cyclophosphamide injected mice sustained substantially less alopecia (Figure 6B, middle and right-hand panels). Histological examination revealed a progression to late catagen in vehicle-treated follicles (Figure 6C left) while EGFR inhibitor treated follicles remained elongated into the subcutis (Figure 6C, middle and right-hand panels), and shared characteristics with *Egfr* mutant follicles (Figure 2). EGFR inhibitor treated hair follicles failed to progress beyond mid-dystrophic catagen following cyclophosphamide (Figure 6C, middle and right-hand panels). As in the *Egfr* mutant mice after cyclophosphamide, vehicle and inhibitor treated mice had similar levels of proliferation as indicated by BrDU (Figure 6D, arrowheads) and PCNA (Figure 6E, arrowheads) immunofluorescence but inhibitor treated mice had fewer p53-positive cells at 3d post-cyclophosphamide (Figure 6F). Thus, EGFR inhibitors administered prior to cyclophosphamide provided partial protection from cyclophosphamide-induced alopecia in mice.

## Discussion

Inhibition or genetic deletion of *Egfr* caused resistance to cyclophosphamide-induced alopecia in the mouse. Cyclophosphamide-treated EGFR deficient follicles entered catagen but did not progress to telogen, as summarized in Figure S5. Since proliferation was reduced in *Egfr* mutant follicles at baseline, less proliferation in the follicle may protect from alopecia. A specific cell cycle defect in cyclophosphamide-treated *Egfr* mutant hair follicles may occur, as indicated by decreased BrDU incorporation but not decreased PCNA labeling after cyclophosphamide. However, no differences were detected in the proliferation of inhibitor compared to vehicle treated follicles after cyclophosphamide. Cyclophosphamide-induced cessation of proliferation is insufficient for alopecia. *Egfr* deletion resulted in partial resistance to apoptosis and p53 up-regulation, a possible underlying mechanism for apoptosis induced by cyclophosphamide.

Cyclophosphamide-induced alopecia results from rapid apoptosis following up-regulation of p53 [15]. Following chemotherapy, cells of the digestive tract and hematopoietic cells also undergo p53-dependent apoptosis [32], [33]. *Trp53* null mice do not respond to cyclophosphamide or undergo apoptosis [15]. p53 induces apoptosis through activation of the Fas receptor, and neutralizing Fas partially inhibits the response to cyclophosphamide [34]. In our study, fewer p53-positive and apoptotic cells occurred in EGFR deficient compared to control follicles after cyclophosphamide. During cyclophosphamide-induced alopecia, initial apoptosis resulting from DNA damage may be independent of EGFR, while sustained induction of apoptosis necessary for hair follicle involution is EGFR-dependent. Alternatively, inefficiency of *Egfr* deletion using Cre/loxP may be a factor. Inhibition of EGFR was also incomplete in our studies.

Our results are consistent with a role for EGFR in stabilization of p53 during cyclophosphamide-induced alopecia. p53 is activated by stress signaling, such as p38 kinase, to activate pro-apoptotic pathways [35]. Thus, EGFR may stabilize p53 through p38 kinase [36]. P38 kinase or Janus kinase (JNK) phosphorylation of p53 delays its degradation, allowing for sustained pro-apoptotic signaling [37]–[39]. Although p38 is detectable in hair follicles [40], its involvement in alopecia is unknown.

Our conclusions about the role of EGFR in cyclophosphamide-induced alopecia were initially derived from a model with conditional deletion of the receptor. Limitations of this model include imperfect excision of *Egfr* by Cre recombinase, as shown in Supplemental Figure 1. In addition, structural defects in *Egfr* mutant follicles could impede chemotherapy-induced apoptosis. In contravention to this idea, however, was the suppression of alopecia by EGFR inhibitors. During dystrophic catagen following exposure to alkylating agents like cyclophosphamide, the hair is shed during the dystrophic catagen phase, a process that was disrupted in the absence of EGFR signaling (see Figure S5 for a schematic summarizing the timing of these events). In mice with intact EGFR function, regrowth of a new hair shaft is initiated during the secondary recovery that follows dystrophic telogen [13]. *Egfr* mutant mice do not complete catagen nor do they undergo alopecia in response to cyclophosphamide. In contrast to the response of the skin to cyclophosphamide, the normal process of exogen during spontaneous hair cycling is independent of the hair cycle and follows telogen when connections to the hair shaft in the follicle are altered [5], [12]. Although *Egfr* deletion disrupts spontaneous catagen [8], [11], the role of the receptor in exogen in the absence of chemical treatment has not been examined.

To examine whether EGFR deficiency decreases alopecia following chemotherapy in patients, secondary analysis of clinical trials employing molecular targeting of EGFR and chemotherapy was undertaken. Less alopecia occurred in five studies, three with small molecule inhibitors erlotinib and gefitinib and two with the antibody cetuximab. One of the trials, using gefitinib plus carboplatin and paclitaxel, reached statistical significance. Nonsignificantly higher rates of alopecia occurred in four EGFR-neutralizing antibody trials. None of the studies with neutralizing antibodies revealed statistically significant differences.

The quality of these analyses is clearly limited by the quality of the data available. Inconsistent reporting of alopecia, i.e. low alopecia rates reported by Herbst et al. (2004), and lack of information about alopecia severity, may have impacted our results. Another key to understanding the lack of consistency among the trials may be the dosing frequencies and half-lives of the EGFR targeted agents (detailed in Table S3). The small molecule inhibitors were administered daily, an interval shorter than their half-lives (T_1/2_ = 50.5 for gefitinib and T_1/2_ = 36.2 hours for erlotinib [41]–[43]). EGFR neutralizing antibodies had longer half-lives, but were administered at an interval longer than their half-lives. Cetuximab has a half-life of 5 days, but was administered weekly, while panitumumab has a half-life of 7.5 days and was administered bi-weekly or every 3 weeks [44]. Thus, small molecule inhibitors would cause sustained inhibition of the receptor while neutralizing antibodies would be intermittent. We hypothesize that sustained inhibition of EGFR is necessary for reduced alopecia. Intermittent inhibition could allow for folliculitis, a common side-effect of all EGFR-targeting agents, without providing protection from alopecia.

Given the folliculitis they can induce that can itself lead to alopecia [45]–[46], a potential protective effect of EGFR inhibitors towards alopecia is surprising. Our results suggest that further research into the role of EGFR signaling in the hair follicle’s response to chemotherapy is warranted. Topical inhibition of signals downstream from EGFR, for example of pro-apoptotic pathways, may allow for protection from chemotherapy-induced alopecia without the folliculitis that results from EGFR inhibition.

## Supporting Information

Figure S1 ***Egfr*** **mutants exhibit decreased EGFR protein compared to controls.** The epidermis was separated from the skin (N = 9 mice/group) by the heat shock method. Immunoblotting of epidermal protein was performed with antibodies recognizing actin (Sigma, St. Louis, MO) or EGFR (Cell Signaling, Danvers, MA) followed by densitometry. *Indicates a significant difference using a Student’s t-test, where P≤0.05.
(TIF)

Figure S2 ***Egfr*** **mutant follicles did not progress through catagen following cyclophosphamide.** Hematoxylin and eosin stained sections from *Egfr* mutant (B) and control (A) mice after cyclophosphamide. Control follicles were in late anagen with elongated follicles and spindle shaped dermal papilla (A) and mutants retained hair 15 d post-administration with no progression to late catagen, telogen or anagen (B). Scale bar indicates 100 µm.
(TIF)

Figure S3 **Quantitative histomorphometry following cyclophosphamide.** Quantitative histomorphometry was performed by light microscopy as described in [12]. Hematoxylin and eosin stained sections from *Egfr* mutants and control mice after cyclophosphamide or vehicle were used to identify hair cycle stage in at least 20 hair follicles per sample and 3 samples per group. MDA =  mid-dystrophic anagen, EDC =  early dystrophic catagen, MDC =  mid-dystrophic catagen, LDC =  late dystrophic catagen, DT =  dystrophic telogen, EA =  early anagen, and LA =  late anagen *Significantly different using a Student’s t-test, where P≤0.05.
(TIF)

Figure S4 **p53 positive immunofluorescence in vehicle treated follicles 2 d post-vehicle.** p53 was similarly localized distal to the hair follicle bulbs of controls (A, above line) and mutants (B) 2 d following vehicle administration with on average fewer than one bulb cell positive in each hair follicle. Scale bar indicates 50 µm.
(TIF)

Figure S5 **Summary of the timing of hair cycle progression and alopecia in** ***Egfr*** **mutant and control mice after cyclophosphamide.** The approximate timing of hair cycle phases and hair loss are shown for *Egfr* mutant (bottom, in red font) and control (middle, in blue font) mice after treatment with cyclophosphamide. For a comparison with spontaneous hair cycling, it is noted that anagen hair follicles typically progress to catagen around P17–P19 while telogen occurs at P20–P21 in the mouse (top, green font).
(TIF)

Table S1 **Search strategy employed for MEDLINE and NIH Clinical Trials databases.**
(DOCX)

Table S2 **Clinical trial inclusion and exclusion criteria.**
(DOCX)

Table S3 **Treatment schedules and dosing for each intervention.**
(DOCX)

Table S4 (DOCX)
