# Supplementary figures and images for: Role for the Epidermal Growth Factor Receptor in Chemotherapy-Induced Alopecia
Source: PLoS One. 2013 Jul 19;8(7):e69368. doi: 10.1371/journal.pone.0069368 (PMC3716704; doi:10.1371/journal.pone.0069368)

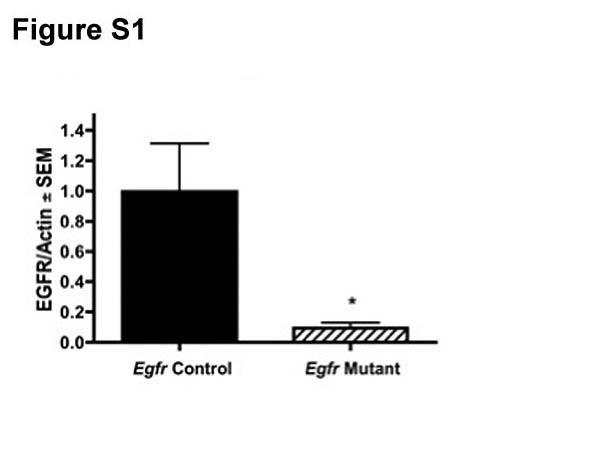

Supplement: Figure S1 — Egfr mutants exhibit decreased EGFR protein compared to controls. The epidermis was separated from the skin (N = 9 mice/group) by the heat shock method. Immunoblotting of epidermal protein was performed with antibodies recognizing actin (Sigma, St. Louis, MO) or EGFR (Cell Signaling, Danvers, MA) followed by densitometry. *Indicates a significant difference using a Student’s t-test, where P≤0.05. (TIF) [file pone.0069368.s001.tif]

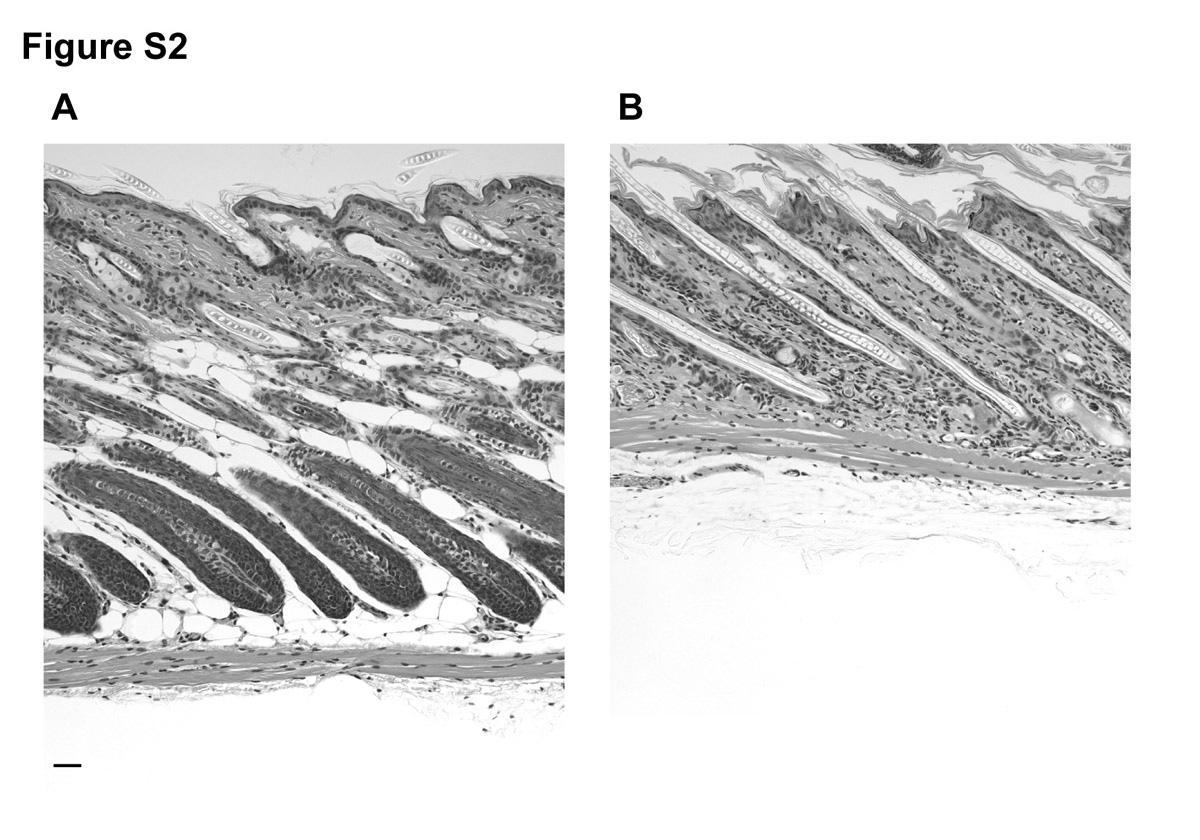

Supplement: Figure S2 — Egfr mutant follicles did not progress through catagen following cyclophosphamide. Hematoxylin and eosin stained sections from Egfr mutant (B) and control (A) mice after cyclophosphamide. Control follicles were in late anagen with elongated follicles and spindle shaped dermal papilla (A) and mutants retained hair 15 d post-administration with no progression to late catagen, telogen or anagen (B). Scale bar indicates 100 µm. (TIF) [file pone.0069368.s002.tif]

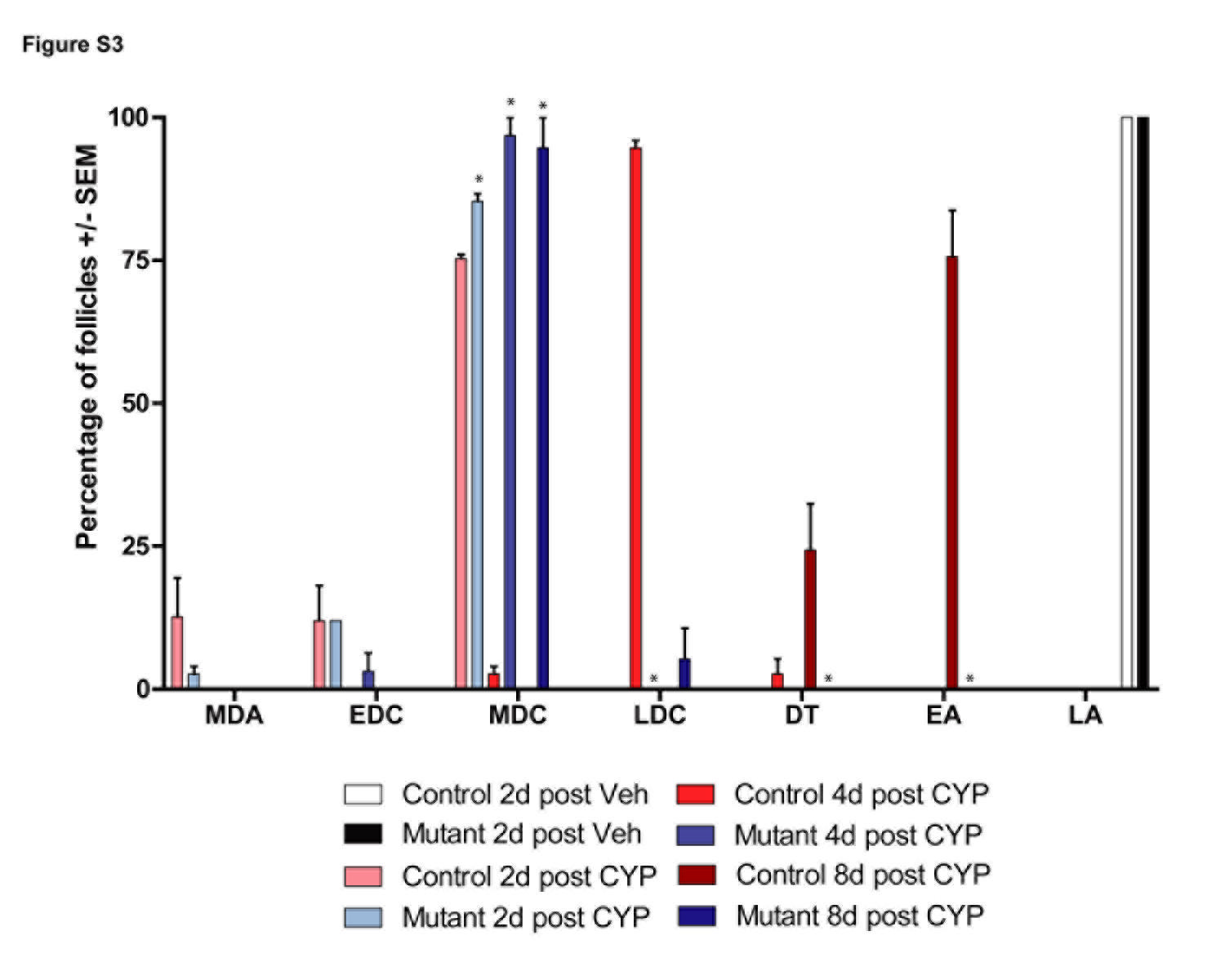

Supplement: Figure S3 — Quantitative histomorphometry following cyclophosphamide. Quantitative histomorphometry was performed by light microscopy as described in [12]. Hematoxylin and eosin stained sections from Egfr mutants and control mice after cyclophosphamide or vehicle were used to identify hair cycle stage in at least 20 hair follicles per sample and 3 samples per group. MDA = mid-dystrophic anagen, EDC = early dystrophic catagen, MDC = mid-dystrophic catagen, LDC = late dystrophic catagen, DT = dystrophic telogen, EA = early anagen, and LA = late anagen *Significantly different using a Student’s t-test, where P≤0.05. (TIF) [file pone.0069368.s003.tif]

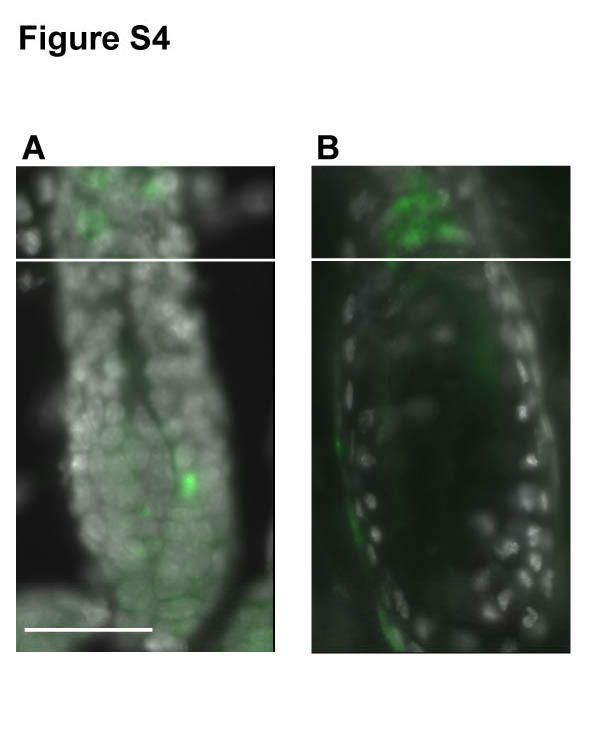

Supplement: Figure S4 — p53 positive immunofluorescence in vehicle treated follicles 2 d post-vehicle. p53 was similarly localized distal to the hair follicle bulbs of controls (A, above line) and mutants (B) 2 d following vehicle administration with on average fewer than one bulb cell positive in each hair follicle. Scale bar indicates 50 µm. (TIF) [file pone.0069368.s004.tif]

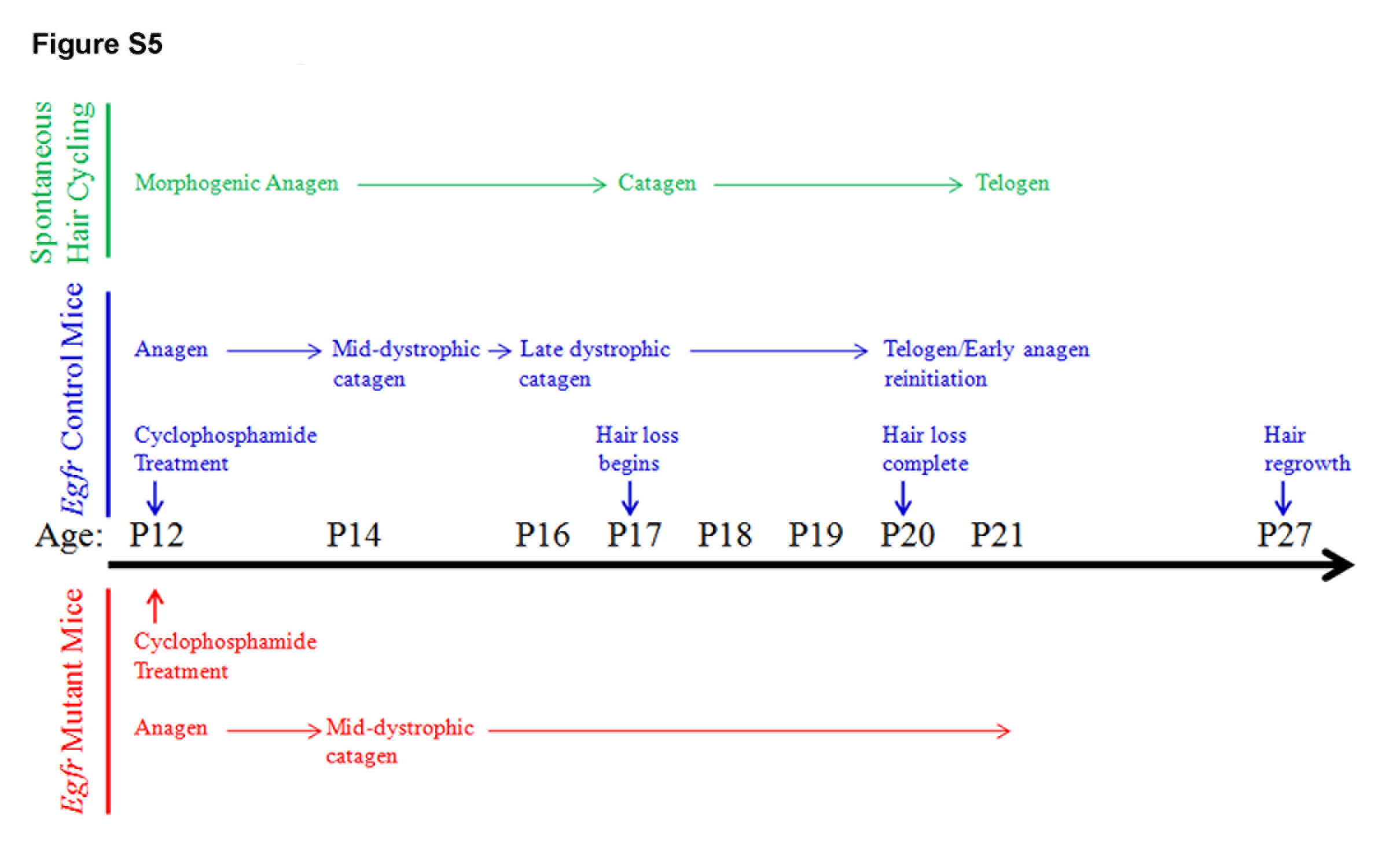

Supplement: Figure S5 — Summary of the timing of hair cycle progression and alopecia in Egfr mutant and control mice after cyclophosphamide. The approximate timing of hair cycle phases and hair loss are shown for Egfr mutant (bottom, in red font) and control (middle, in blue font) mice after treatment with cyclophosphamide. For a comparison with spontaneous hair cycling, it is noted that anagen hair follicles typically progress to catagen around P17–P19 while telogen occurs at P20–P21 in the mouse (top, green font). (TIF) [file pone.0069368.s005.tif]
