# Supplementary material for: Role for the Epidermal Growth Factor Receptor in Chemotherapy-Induced Alopecia
Source: PLoS One. 2013 Jul 19;8(7):e69368. doi: 10.1371/journal.pone.0069368 (PMC3716704; doi:10.1371/journal.pone.0069368)
Supplement: Table S1 — Search strategy employed for MEDLINE and NIH Clinical Trials databases. (DOCX) [file pone.0069368.s006.docx]

**Table S1. Search strategy employed for MEDLINE and NIH Clinical Trials databases.**

| **Search Terms^1^** | |
| --- | --- |
| Antineoplastic agents, EGFR Inhibitor | Cetuximab, Bleomycin |
| EGFR Inhibitor, Bleomycin | Cetuximab, Cyclophosphamide |
| EGFR Inhibitor, Cyclophosphamide | Cetuximab, Dactinomycin |
| EGFR Inhibitor, Dactinomycin | Cetuximab, Doxorubicin |
| EGFR Inhibitor, Doxorubicin | Cetuximab, Irinotecan |
| EGFR Inhibitor, Irinotecan | Cetuximab, Paclitaxel |
| EGFR Inhibitor, Paclitaxel | Cetuximab, Topotecan |
| EGFR Inhibitor, Topotecan | Panitumumab, Bleomycin |
| Erlotinib, Bleomycin | Panitumumab, Cyclophosphamide |
| Erlotinib, Cyclophosphamide | Panitumumab, Dactinomycin |
| Erlotinib, Dactinomycin | Panitumumab, Doxorubicin |
| Erlotinib, Doxorubicin | Panitumumab, Irinotecan |
| Erlotinib, Irinotecan | Panitumumab, Paclitaxel |
| Erlotinib, Paclitaxel | Panitumumab, Topotecan |
| Erlotinib, Topotecan |  |
| Gefitinib, Bleomycin |  |
| Gefitinib, Cyclophosphamide |  |
| Gefitinib, Dactinomycin |  |
| Gefitinib, Doxorubicin |  |
| Gefitinib, Irinotecan |  |
| Gefitinib, Paclitaxel |  |
| Gefitinib, Topotecan |  |

^1^Each cell represents 1 search
