# Supplementary material for: Role for the Epidermal Growth Factor Receptor in Chemotherapy-Induced Alopecia
Source: PLoS One. 2013 Jul 19;8(7):e69368. doi: 10.1371/journal.pone.0069368 (PMC3716704; doi:10.1371/journal.pone.0069368)
Supplement: Table S2 — Clinical trial inclusion and exclusion criteria. (DOCX) [file pone.0069368.s007.docx]

**Table S2. Clinical trial inclusion and exclusion criteria.**

| **Inclusion** | **Exclusion** |
| --- | --- |
| Chemotherapy used known to induce alopecia | EGFR targeted therapy plus chemotherapy without matched control groups |
| Trials with control group (group not administered EGFR targeted therapy) | Treatments that did not include EGFR targeted therapy plus alopecia-inducing chemotherapy |
| EGFR-targeted therapy administered concurrently or prior to chemotherapy | Dual specificity EGFR-targeted therapy i.e Lapatinib (EGFR and ErbB2 inhibitor) |
| Patients were randomized to treatment arms | Non-specific small molecule inhibitors used i.e non-specific tyrosine kinase inhibitors |
| Alopecia rates reported | EGFR-targeted therapy not administered concurrently or prior to chemotherapy |
| Complete description of treatment schedules provided | Alopecia rates not reported |
|  | Trials including radiation |
|  | Trials published in languages other than English |
|  | Trials containing fewer than 10 participants |
