# Supplementary material for: Role for the Epidermal Growth Factor Receptor in Chemotherapy-Induced Alopecia
Source: PLoS One. 2013 Jul 19;8(7):e69368. doi: 10.1371/journal.pone.0069368 (PMC3716704; doi:10.1371/journal.pone.0069368)
Supplement: Table S3 — Treatment schedules and dosing for each intervention. (DOCX) [file pone.0069368.s008.docx]

**Table S3. Treatment schedules and dosing for each intervention.**

| Herbst et al. 2004 [25] | Gefitinib: 250 mg/d or 500 mg/d (Maintained until disease progression) |  |
| --- | --- | --- |
|  | Paclitaxel: 225 mg/m2 on day 1 every 3 wks 6 cycles |  |
|  | Carboplatin: AUC 6 on day 1 every 3 wks 6 cycles |  |
| Guarneri et al. 2008[26] | Gefitinib: 250 mg/d (Day 1 to Day 21) |  |
|  | Epirubicin: 90 mg/m2 on day 1 every 3 wks 4 cycles |  |
|  | Paclitaxel: 175 mg/m2 on day 1 every 3 wks 4 cycles |  |
| Herbst et al. 2005 [27] | Erlotinib: 150 mg/d |  |
|  | Paclitaxel: 200 mg/m2 on day 1 every 3 wks 6 cycles |  |
|  | Carboplatin: AUC 6 on day 1 every 3 wks 6 cycles |  |
| NCT00154102, Van Cutsem et al. 2009 [28] | Cetuximab: 400 mg/m2 (initial), 250 mg/m2 (weekly) until disease progression |  |
|  | FOLFIRI: Biweekly |  |
|  | Irinotecan: 180 mg/m2 |  |
|  | Leucovorin: 400 mg/m2 or 200mg/m2 |  |
|  | 5-FU: 400mg/m2 bolus followed by 46 hr. continuous 2400 mg/m2 |  |
| NCT00042939 | Cetuximab: 400 mg/m2 (initial), 250 mg/m2 (weekly) |  |
|  | Docetaxel: 35 mg/m2 (weekly for 4 wks, then 2 wks off) |  |
|  | Irinotecan: 35mg/m2 (weekly for 4 wks, then 2 wks off) |  |
| NCT00148798, Pirker et al. 2009 [29] | Cetuximab: 400 mg/m2 (initial), 250 mg/m2 (weekly) until disease progression |  |
|  | Cisplatin: 80 mg/m2 on day 1 every 3 wks |  |
|  | Vinorelbine: 25 mg/m2 on day 1 and 8 every 3 wks |  |
| NCT00122460, Vermorken et al. 2008 [30] | Cetuximab: 400 mg/m2 (initial), 250 mg/m2 (weekly) |  |
|  | Cisplatin: 100 mg/m2 on day 1 every 3 wks | Chemo given as Cisplatin + 5-FU or Carboplatin + 5-FU |
|  | 5-FU: 1000 mg/m2 continuous from day 1-4 every 3 wks |  |
|  | Carboplatin: AUC 5 on day 1 every 3 wks |  |
| NCT00115765, Hecht et al. 2009 [31] | Panitumumab: 6 mg/kg, day 1 every 2 wks |  |
|  | Oxaliplatin + 5-FU + Leucovorin, day 1 every 2 wks |  |
|  | Irinotecan + 5-FU + Leucovorin, day 1 every 2 wks |  |
| Okines et al 2010 [32] | Panitumumab: 9 mg/kg, day 1 every 3 wks |  |
|  | Epirubicin: 50 mg/m2 every 3 wks | Dose de-escalation groups also used |
|  | Oxaliplatin: 130 mg/m2 every 3 wks |  |
|  | Capecitibine: 1000 mg/m2 every 3 wks |  |
