# Supplementary material for: Role for the Epidermal Growth Factor Receptor in Chemotherapy-Induced Alopecia
Source: PLoS One. 2013 Jul 19;8(7):e69368. doi: 10.1371/journal.pone.0069368 (PMC3716704; doi:10.1371/journal.pone.0069368)
Supplement: Table S4 — (DOCX) [file pone.0069368.s009.docx]

| Search Term (NIH Clinical Trials) | Trial Excluded | Reason for Exclusion |
| --- | --- | --- |
| EGFR inhibitor antineoplastic agents | NCT00054275 | No control group |
| EGFR inhibitor antineoplastic agents | NCT00479856 | No control group |
| EGFR inhibitor antineoplastic agents | NCT00418886 | Alopecia rates not reported |
| EGFR inhibitor antineoplastic agents | NCT00371566 | Alopecia rates not reported |
| EGFR inhibitor antineoplastic agents | NCT00312377 | Non-specific EGFR inhibitor used |
| EGFR inhibitor antineoplastic agents | NCT00294762 | No control group |
| EGFR inhibitor antineoplastic agents | NCT00265317 | No control group |
| EGFR Inhibitor Bleomycin | None |  |
| EGFR Inhibitor Cyclophosphamide | None |  |
| EGFR Inhibitor Dactinomycin | None |  |
| EGFR Inhibitor Doxorubicin | None |  |
| EGFR Inhibitor Irinotecan | None |  |
| EGFR Inhibitor Paclitaxel | NCT00063258 | Less than 10 participants |
| EGFR Inhibitor Topotecan | None |  |
| Erlotinib Bleomycin | None |  |
| Erlotinib Cyclophosphamide | None |  |
| Erlotinib Dactinomycin | None |  |
| Erlotinib Doxorubicin | None |  |
| Erlotinib Irinotecan | None |  |
| Erlotinib Paclitaxel | NCT00085839 | No control group |
| Erlotinib Topotecan | NCT00611468 | No control group |
| Gefitinib Bleomycin | None |  |
| Gefitinib Cyclophosphamide | None |  |
| Gefitinib Dactinomycin | None |  |
| Gefitinib Doxorubicin | None |  |
| Gefitinib Irinotecan | None |  |
| Gefitinib Paclitaxel | None |  |
| Gefitinib Topotecan | None |  |
| Cetuximab Bleomycin | None |  |
| Cetuximab Cyclophosphamide | None |  |
| Cetuximab Dactinomycin | None |  |
| Cetuximab Doxorubicin | None |  |
| Cetuximab Irinotecan | NCT00110357 | No control group |
| Cetuximab Irinotecan | NCT00677924 | No control group |
| Cetuximab Paclitaxel | NCT00343291 | No control group |
| Cetuximab Paclitaxel | NCT00815308 | No control group |
| Cetuximab Topotecan | None |  |
| Panitumumab Bleomycin | None |  |
| Panitumumab Cyclophosphamide | None |  |
| Panitumumab Dactinomycin | None |  |
| Panitumumab Doxorubicin | None |  |
| Panitumumab Irinotecan | NCT00111761 | No control group |
| Panitumumab Paclitaxel | None |  |
| Panitumumab Topotecan | None |  |

| Search Term (MEDLINE) | Trial Excluded | Reason for Exclusion |
| --- | --- | --- |
| EGFR inhibitor antineoplastic agents | [Phase II study of docetaxel and gefitinib as second-line therapy in gemcitabine pretreated patients with advanced pancreatic cancer.](http://www.ncbi.nlm.nih.gov/pubmed/19258727) | No control group |
|  | Brell JM, Matin K, Evans T, Volkin RL, Kiefer GJ, Schlesselman JJ, Dranko S, Rath L, Schmotzer A, Lenzner D, Ramanathan RK. |  |
|  | Oncology. 2009;76(4):270-4. Epub 2009 Mar 4. |  |
|  |  |  |
| EGFR inhibitor antineoplastic agents | [Cetuximab, topotecan and cisplatin for the treatment of advanced cervical cancer: A phase II GINECO trial.](http://www.ncbi.nlm.nih.gov/pubmed/19232434) | No control group |
|  | Kurtz JE, Hardy-Bessard AC, Deslandres M, Lavau-Denes S, Largillier R, Roemer-Becuwe C, Weber B, Guillemet C, Paraiso D, Pujade-Lauraine E. |  |
|  | Gynecol Oncol. 2009 Apr;113(1):16-20. Epub 2009 Feb 15 |  |
|  |  |  |
| EGFR inhibitor antineoplastic agents | [Erlotinib and bevacizumab in patients with recurrent or metastatic squamous-cell carcinoma of the head and neck: a phase I/II study.](http://www.ncbi.nlm.nih.gov/pubmed/19201650) | No control group |
|  | Cohen EE, Davis DW, Karrison TG, Seiwert TY, Wong SJ, Nattam S, Kozloff MF, Clark JI, Yan DH, Liu W, Pierce C, Dancey JE, Stenson K, Blair E, Dekker A, Vokes EE. |  |
|  | Lancet Oncol. 2009 Mar;10(3):247-57. Epub 2009 Feb 7. |  |
|  |  |  |
| EGFR inhibitor antineoplastic agents | [Phase I/II trial of erlotinib and temozolomide with radiation therapy in the treatment of newly diagnosed glioblastoma multiforme: North Central Cancer Treatment Group Study N0177.](http://www.ncbi.nlm.nih.gov/pubmed/18955445) | Use of radiation |
|  | Brown PD, Krishnan S, Sarkaria JN, Wu W, Jaeckle KA, Uhm JH, Geoffroy FJ, Arusell R, Kitange G, Jenkins RB, Kugler JW, Morton RF, Rowland KM Jr, Mischel P, Yong WH, Scheithauer BW, Schiff D, Giannini C, Buckner JC; North Central Cancer Treatment Group Study N0177. |  |
|  | J Clin Oncol. 2008 Dec 1;26(34):5603-9. Epub 2008 Oct 27. |  |
|  |  |  |
| EGFR inhibitor antineoplastic agents | [Pilot study of gefitinib, oxaliplatin, and radiotherapy for esophageal adenocarcinoma: tissue effect predicts clinical response.](http://www.ncbi.nlm.nih.gov/pubmed/18845990) | No control group |
|  | Javle M, Pande A, Iyer R, Yang G, LeVea C, Wilding G, Black J, Nava H, Nwogu C. |  |
|  | Am J Clin Oncol. 2008 Aug;31(4):329-34. |  |
|  |  |  |
| EGFR inhibitor antineoplastic agents | [Pilot study of gefitinib and fulvestrant in the treatment of post-menopausal women with advanced non-small cell lung cancer.](http://www.ncbi.nlm.nih.gov/pubmed/18701186) | No control group |
|  | Traynor AM, Schiller JH, Stabile LP, Kolesar JM, Eickhoff JC, Dacic S, Hoang T, Dubey S, Marcotte SM, Siegfried JM. |  |
|  | Lung Cancer. 2009 Apr;64(1):51-9. Epub 2008 Aug 12. |  |
|  |  |  |
| EGFR inhibitor antineoplastic agents | [A phase II trial of gefitinib in patients with non-metastatic hormone-refractory prostate cancer.](http://www.ncbi.nlm.nih.gov/pubmed/17822457) | No concurrent chemotherapy |
|  | Small EJ, Fontana J, Tannir N, DiPaola RS, Wilding G, Rubin M, Iacona RB, Kabbinavar FF. |  |
|  | BJU Int. 2007 Oct;100(4):765-9. |  |
|  |  |  |
| EGFR inhibitor antineoplastic agents | [A phase II placebo-controlled trial of neoadjuvant anastrozole alone or with gefitinib in early breast cancer.](http://www.ncbi.nlm.nih.gov/pubmed/17679728) | No control group |
|  | Smith IE, Walsh G, Skene A, Llombart A, Mayordomo JI, Detre S, Salter J, Clark E, Magill P, Dowsett M. |  |
|  | J Clin Oncol. 2007 Sep 1;25(25):3816-22. Epub 2007 Aug 6. |  |
|  |  |  |
| EGFR inhibitor antineoplastic agents | [Phase I study of EKB-569, an irreversible inhibitor of the epidermal growth factor receptor, in patients with advanced solid tumors.](http://www.ncbi.nlm.nih.gov/pubmed/16710023) | No concurrent chemotherapy |
|  | Erlichman C, Hidalgo M, Boni JP, Martins P, Quinn SE, Zacharchuk C, Amorusi P, Adjei AA, Rowinsky EK. |  |
|  | J Clin Oncol. 2006 May 20;24(15):2252-60. |  |
|  |  |  |
| EGFR inhibitor antineoplastic agents | [Increased toxicity with gefitinib, capecitabine, and radiation therapy in pancreatic and rectal cancer: phase I trial results.](http://www.ncbi.nlm.nih.gov/pubmed/16446337) | Use of radiation |
|  | Czito BG, Willett CG, Bendell JC, Morse MA, Tyler DS, Fernando NH, Mantyh CR, Blobe GC, Honeycutt W, Yu D, Clary BM, Pappas TN, Ludwig KA, Hurwitz HI. |  |
|  | J Clin Oncol. 2006 Feb 1;24(4):656-62. |  |
|  |  |  |
| EGFR inhibitor antineoplastic agents | [Multicenter phase I/II study of cetuximab with paclitaxel and carboplatin in untreated patients with stage IV non-small-cell lung cancer.](http://www.ncbi.nlm.nih.gov/pubmed/16246975) | No control group |
|  | Thienelt CD, Bunn PA Jr, Hanna N, Rosenberg A, Needle MN, Long ME, Gustafson DL, Kelly K. |  |
|  | J Clin Oncol. 2005 Dec 1;23(34):8786-93. Epub 2005 Oct 24. |  |
|  |  |  |
| EGFR inhibitor antineoplastic agents | [Phase I and pharmacokinetic study of BIBX 1382 BS, an epidermal growth factor receptor (EGFR) inhibitor, given in a continuous daily oral administration.](http://www.ncbi.nlm.nih.gov/pubmed/12008195) | No concurrent chemotherapy |
|  | Dittrich Ch, Greim G, Borner M, Weigang-Köhler K, Huisman H, Amelsberg A, Ehret A, Wanders J, Hanauske A, Fumoleau P. |  |
|  | Eur J Cancer. 2002 May;38(8):1072-80. |  |
| EGFR Inhibitor Bleomycin | None |  |
| EGFR Inhibitor Cyclophosphamide | None |  |
| EGFR Inhibitor Dactinomycin | None |  |
| EGFR Inhibitor Doxorubicin | None |  |
| EGFR Inhibitor Irinotecan | [Does erlotinib restore chemosensitivity to chemotherapy in pancreatic cancer? A case series.](http://www.ncbi.nlm.nih.gov/pubmed/21498735) | No control group, case series |
|  | Saif MW. |  |
|  | Anticancer Res. 2011 Mar;31(3):1039-42. |  |
|  |  |  |
| EGFR Inhibitor Irinotecan | [Phase I pharmacokinetic/pharmacodynamic study of EKB-569, an irreversible inhibitor of the epidermal growth factor receptor tyrosine kinase, in combination with irinotecan, 5-fluorouracil, and leucovorin (FOLFIRI) in first-line treatment of patients with metastatic colorectal cancer.](http://www.ncbi.nlm.nih.gov/pubmed/18172273) | No control group |
|  | Folprecht G, Tabernero J, Köhne CH, Zacharchuk C, Paz-Ares L, Rojo F, Quinn S, Casado E, Salazar R, Abbas R, Lejeune C, Marimón I, Andreu J, Ubbelohde U, Cortes-Funes H, Baselga J. |  |
|  | Clin Cancer Res. 2008 Jan 1;14(1):215-23. |  |
|  |  |  |
| EGFR Inhibitor Irinotecan | [Phase I study of gefitinib plus FOLFIRI in previously untreated patients with metastatic colorectal cancer.](http://www.ncbi.nlm.nih.gov/pubmed/17026790) | No control group |
|  | Wolpin BM, Clark JW, Meyerhardt JA, Earle CC, Ryan DP, Enzinger PC, Zhu AX, Blaszkowsky L, Battu S, Fuchs CS. |  |
|  | Clin Colorectal Cancer. 2006 Sep;6(3):208-13. |  |
|  |  |  |
| EGFR Inhibitor Irinotecan | [A phase II trial of gefitinib with 5-fluorouracil, leucovorin, and irinotecan in patients with colorectal cancer.](http://www.ncbi.nlm.nih.gov/pubmed/15870719) | No control group |
|  | Veronese ML, Sun W, Giantonio B, Berlin J, Shults J, Davis L, Haller DG, O'Dwyer PJ. |  |
|  | Br J Cancer. 2005 May 23;92(10):1846-9. |  |
| EGFR inhibitor, Paclitaxel | [A single-arm phase II trial of first-line paclitaxel in combination with lapatinib in HER2-overexpressing metastatic breast cancer.](http://www.ncbi.nlm.nih.gov/pubmed/21088439) | No control group |
|  | Jagiello-Gruszfeld A, Tjulandin S, Dobrovolskaya N, Manikhas A, Pienkowski T, DeSilvio M, Ridderheim M, Abbey R. |  |
|  | Oncology. 2010;79(1-2):129-35. Epub 2010 Nov 22. |  |
|  |  |  |
| EGFR inhibitor, Paclitaxel | [Erlotinib added to carboplatin and paclitaxel as first-line treatment of ovarian cancer: a phase II study based on surgical reassessment.](http://www.ncbi.nlm.nih.gov/pubmed/20837357) | No control group |
|  | Blank SV, Christos P, Curtin JP, Goldman N, Runowicz CD, Sparano JA, Liebes L, Chen HX, Muggia FM. |  |
|  | Gynecol Oncol. 2010 Dec;119(3):451-6. Epub 2010 Sep 15. |  |
|  |  |  |
| EGFR inhibitor, Paclitaxel | [Epidermal growth factor receptor inhibitor gefitinib added to chemoradiotherapy in locally advanced head and neck cancer.](http://www.ncbi.nlm.nih.gov/pubmed/20498391) | No control group |
|  | Cohen EE, Haraf DJ, Kunnavakkam R, Stenson KM, Blair EA, Brockstein B, Lester EP, Salama JK, Dekker A, Williams R, Witt ME, Grushko TA, Dignam JJ, Lingen MW, Olopade OI, Vokes EE. |  |
|  | J Clin Oncol. 2010 Jul 10;28(20):3336-43. Epub 2010 May 24. |  |
|  |  |  |
| EGFR inhibitor, Paclitaxel | [Molecular and clinical responses in a pilot study of gefitinib with paclitaxel and radiation in locally advanced head-and-neck cancer.](http://www.ncbi.nlm.nih.gov/pubmed/19879702) | No control group |
|  | Van Waes C, Allen CT, Citrin D, Gius D, Colevas AD, Harold NA, Rudy S, Nottingham L, Muir C, Chen Z, Singh AK, Dancey J, Morris JC. |  |
|  | Int J Radiat Oncol Biol Phys. 2010 Jun 1;77(2):447-54. Epub 2009 Oct 30. |  |
|  |  |  |
| EGFR inhibitor, Paclitaxel | [Estrogen receptor, progesterone receptor, human epidermal growth factor receptor 2 (HER2), and epidermal growth factor receptor expression and benefit from lapatinib in a randomized trial of paclitaxel with lapatinib or placebo as first-line treatment in HER2-negative or unknown metastatic breast cancer.](http://www.ncbi.nlm.nih.gov/pubmed/19620495) | Non-specific EGFR inhibitor |
|  | Finn RS, Press MF, Dering J, Arbushites M, Koehler M, Oliva C, Williams LS, Di Leo A. |  |
|  | J Clin Oncol. 2009 Aug 20;27(24):3908-15. Epub 2009 Jul 20. |  |
|  |  |  |
| EGFR inhibitor, Paclitaxel | [Phase III, double-blind, randomized study comparing lapatinib plus paclitaxel with placebo plus paclitaxel as first-line treatment for metastatic breast cancer.](http://www.ncbi.nlm.nih.gov/pubmed/18955454) | Non-specific EGFR inhibitor |
|  | Di Leo A, Gomez HL, Aziz Z, Zvirbule Z, Bines J, Arbushites MC, Guerrera SF, Koehler M, Oliva C, Stein SH, Williams LS, Dering J, Finn RS, Press MF. |  |
|  | J Clin Oncol. 2008 Dec 1;26(34):5544-52. Epub 2008 Oct 27. Erratum in: J Clin Oncol. 2009 Apr 10;27(11):1923. |  |
|  |  |  |
| EGFR inhibitor, Paclitaxel | [Phase I, pharmacokinetic, and biological study of erlotinib in combination with paclitaxel and carboplatin in patients with advanced solid tumors.](http://www.ncbi.nlm.nih.gov/pubmed/17189413) | No control group |
|  | Patnaik A, Wood D, Tolcher AW, Hamilton M, Kreisberg JI, Hammond LA, Schwartz G, Beeram M, Hidalgo M, Mita MM, Wolf J, Nadler P, Rowinsky EK. |  |
|  | Clin Cancer Res. 2006 Dec 15;12(24):7406-13. |  |
|  |  |  |
| EGFR inhibitor, Paclitaxel | [Multicenter phase I/II study of cetuximab with paclitaxel and carboplatin in untreated patients with stage IV non-small-cell lung cancer.](http://www.ncbi.nlm.nih.gov/pubmed/16246975) | No control group |
|  | Thienelt CD, Bunn PA Jr, Hanna N, Rosenberg A, Needle MN, Long ME, Gustafson DL, Kelly K. |  |
|  | J Clin Oncol. 2005 Dec 1;23(34):8786-93. Epub 2005 Oct 24. |  |
|  |  |  |
| EGFR inhibitor, Paclitaxel | [Cetuximab, topotecan and cisplatin for the treatment of advanced cervical cancer: A phase II GINECO trial.](http://www.ncbi.nlm.nih.gov/pubmed/19232434) | No control group |
|  | Kurtz JE, Hardy-Bessard AC, Deslandres M, Lavau-Denes S, Largillier R, Roemer-Becuwe C, Weber B, Guillemet C, Paraiso D, Pujade-Lauraine E. |  |
|  | Gynecol Oncol. 2009 Apr;113(1):16-20. Epub 2009 Feb 15. |  |
|  |  |  |
| EGFR inhibitor, Paclitaxel | [Phase II trial of lapatinib and topotecan (LapTop) in patients with platinum-refractory/resistant ovarian and primary peritoneal carcinoma.](http://www.ncbi.nlm.nih.gov/pubmed/21514634) | No control group |
|  | Weroha SJ, Oberg AL, Ziegler KL, Dakhilm SR, Rowland KM, Hartmann LC, Moore DF Jr, Keeney GL, Peethambaram PP, Haluska P. |  |
|  | Gynecol Oncol. 2011 Jul;122(1):116-20. Epub 2011 Apr 22. |  |
| Erlotinib, Bleomycin | None |  |
| Erlotinib, Cyclophosphamide | None |  |
| Erlotinib, Dactinomycin | None |  |
| Erlotinib, Doxorubicin | [Treatment of advanced adrenocortical carcinoma with erlotinib plus gemcitabine.](http://www.ncbi.nlm.nih.gov/pubmed/18334586) | No control group |
|  | Quinkler M, Hahner S, Wortmann S, Johanssen S, Adam P, Ritter C, Strasburger C, Allolio B, Fassnacht M. |  |
|  | J Clin Endocrinol Metab. 2008 Jun;93(6):2057-62. Epub 2008 Mar 11. |  |
| Erlotinib, Irinotecan | [Dose finding study of erlotinib combined to capecitabine and irinotecan in pretreated advanced colorectal cancer patients.](http://www.ncbi.nlm.nih.gov/pubmed/18936940) | No control group |
|  | Bajetta E, Di Bartolomeo M, Buzzoni R, Ferrario E, Dotti KF, Mariani L, Bajetta R, Gevorgyan A, Venturino P, Galassi M. |  |
|  | Cancer Chemother Pharmacol. 2009 Jun;64(1):67-72. Epub 2008 Oct 21. |  |
|  |  |  |
| Erlotinib, Irinotecan | [A phase Ib dose-escalation study of erlotinib, capecitabine and oxaliplatin in metastatic colorectal cancer patients.](http://www.ncbi.nlm.nih.gov/pubmed/17986625) | Anti-EGFR administered following chemotherapy |
|  | Van Cutsem E, Verslype C, Beale P, Clarke S, Bugat R, Rakhit A, Fettner SH, Brennscheidt U, Feyereislova A, Delord JP. |  |
|  | Ann Oncol. 2008 Feb;19(2):332-9. Epub 2007 Nov 6. |  |
|  |  |  |
| Erlotinib, Irinotecan | [Phase II study of capecitabine, oxaliplatin, and erlotinib in previously treated patients with metastastic colorectal cancer.](http://www.ncbi.nlm.nih.gov/pubmed/16622264) | No control group |
|  | Meyerhardt JA, Zhu AX, Enzinger PC, Ryan DP, Clark JW, Kulke MH, Earle CC, Vincitore M, Michelini A, Sheehan S, Fuchs CS. |  |
|  | J Clin Oncol. 2006 Apr 20;24(12):1892-7. |  |
|  |  |  |
| Erlotinib, Irinotecan | [Phase I trial of irinotecan, infusional 5-fluorouracil, and leucovorin (FOLFIRI) with erlotinib (OSI-774): early termination due to increased toxicities.](http://www.ncbi.nlm.nih.gov/pubmed/15475439) | No control group |
|  | Messersmith WA, Laheru DA, Senzer NN, Donehower RC, Grouleff P, Rogers T, Kelley SK, Ramies DA, Lum BL, Hidalgo M. |  |
|  | Clin Cancer Res. 2004 Oct 1;10(19):6522-7. |  |
| Erlotinib, Paclitaxel | [Targeted and cytotoxic therapy in coordinated sequence (TACTICS): erlotinib, bevacizumab, and standard chemotherapy for non-small-cell lung cancer, a phase II trial.](http://www.ncbi.nlm.nih.gov/pubmed/22100149) | No control group |
|  | Cohen EE, Subramanian J, Gao F, Szeto L, Kozloff M, Faoro L, Karrison T, Salgia R, Govindan R, Vokes EE. |  |
|  | Clin Lung Cancer. 2012 Mar;13(2):123-8. Epub 2011 Nov 18. |  |
|  |  |  |
| Erlotinib, Paclitaxel | [Combined modality treatment with chemotherapy, radiation therapy, bevacizumab, and erlotinib in patients with locally advanced squamous carcinoma of the head and neck: a phase II trial of the Sarah Cannon oncology research consortium.](http://www.ncbi.nlm.nih.gov/pubmed/21952273) | No control group, Anti-EGFR administered following chemotherapy |
|  | Hainsworth JD, Spigel DR, Greco FA, Shipley DL, Peyton J, Rubin M, Stipanov M, Meluch A. |  |
|  | Cancer J. 2011 Sep-Oct;17(5):267-72. |  |
|  |  |  |
| Erlotinib, Paclitaxel | [A randomized, phase II, biomarker-selected study comparing erlotinib to erlotinib intercalated with chemotherapy in first-line therapy for advanced non-small-cell lung cancer.](http://www.ncbi.nlm.nih.gov/pubmed/21825259) | Alopeica rates not reported |
|  | Hirsch FR, Kabbinavar F, Eisen T, Martins R, Schnell FM, Dziadziuszko R, Richardson K, Richardson F, Wacker B, Sternberg DW, Rusk J, Franklin WA, Varella-Garcia M, Bunn PA Jr, Camidge DR. |  |
|  | J Clin Oncol. 2011 Sep 10;29(26):3567-73. Epub 2011 Aug 8. Erratum in: J Clin Oncol. 2011 Oct 10;29(29):3948. |  |
|  |  |  |
| Erlotinib, Paclitaxel | [Phase II selection design trial of concurrent chemotherapy and cetuximab versus chemotherapy followed by cetuximab in advanced-stage non-small-cell lung cancer: Southwest Oncology Group study S0342.](http://www.ncbi.nlm.nih.gov/pubmed/20921467) | No control group |
|  | Herbst RS, Kelly K, Chansky K, Mack PC, Franklin WA, Hirsch FR, Atkins JN, Dakhil SR, Albain KS, Kim ES, Redman M, Crowley JJ, Gandara DR. |  |
|  | J Clin Oncol. 2010 Nov 1;28(31):4747-54. Epub 2010 Oct 4. |  |
|  |  |  |
| Erlotinib, Paclitaxel | [Erlotinib added to carboplatin and paclitaxel as first-line treatment of ovarian cancer: a phase II study based on surgical reassessment.](http://www.ncbi.nlm.nih.gov/pubmed/20837357) | No control group |
|  | Blank SV, Christos P, Curtin JP, Goldman N, Runowicz CD, Sparano JA, Liebes L, Chen HX, Muggia FM. |  |
|  | Gynecol Oncol. 2010 Dec;119(3):451-6. Epub 2010 Sep 15. |  |
|  |  |  |
| Erlotinib, Paclitaxel | [Phase II study of concurrent chemoradiation in combination with erlotinib for locally advanced esophageal carcinoma.](http://www.ncbi.nlm.nih.gov/pubmed/20350790) | No control group |
|  | Li G, Hu W, Wang J, Deng X, Zhang P, Zhang X, Xie C, Wu S. |  |
|  | Int J Radiat Oncol Biol Phys. 2010 Dec 1;78(5):1407-12. Epub 2010 Mar 28. |  |
|  |  |  |
| Erlotinib, Paclitaxel | [Paclitaxel/carboplatin plus bevacizumab/erlotinib in the first-line treatment of patients with carcinoma of unknown primary site.](http://www.ncbi.nlm.nih.gov/pubmed/19965914) | No control group |
|  | Hainsworth JD, Spigel DR, Thompson DS, Murphy PB, Lane CM, Waterhouse DM, Naot Y, Greco FA. |  |
|  | Oncologist. 2009 Dec;14(12):1189-97. Epub 2009 Dec 4. |  |
|  |  |  |
| Erlotinib, Paclitaxel | [Randomized phase II study of pulse erlotinib before or after carboplatin and paclitaxel in current or former smokers with advanced non-small-cell lung cancer.](http://www.ncbi.nlm.nih.gov/pubmed/19047285) | Anti-EGFR administered following chemotherapy |
|  | Riely GJ, Rizvi NA, Kris MG, Milton DT, Solit DB, Rosen N, Senturk E, Azzoli CG, Brahmer JR, Sirotnak FM, Seshan VE, Fogle M, Ginsberg M, Miller VA, Rudin CM. |  |
|  | J Clin Oncol. 2009 Jan 10;27(2):264-70. Epub 2008 Dec 1. |  |
|  |  |  |
| Erlotinib, Paclitaxel | [Phase I trial of erlotinib-based multimodality therapy for inoperable stage III non-small cell lung cancer.](http://www.ncbi.nlm.nih.gov/pubmed/18758303) | Anti-EGFR administered following chemotherapy |
|  | Choong NW, Mauer AM, Haraf DJ, Lester E, Hoffman PC, Kozloff M, Lin S, Dancey JE, Szeto L, Grushko T, Olopade OI, Salgia R, Vokes EE. |  |
|  | J Thorac Oncol. 2008 Sep;3(9):1003-11. |  |
|  |  |  |
| Erlotinib, Paclitaxel | [Randomized phase II trial of erlotinib or standard chemotherapy in patients with advanced non-small-cell lung cancer and a performance status of 2.](http://www.ncbi.nlm.nih.gov/pubmed/18281658) | No concurrent chemotherapy |
|  | Lilenbaum R, Axelrod R, Thomas S, Dowlati A, Seigel L, Albert D, Witt K, Botkin D. |  |
|  | J Clin Oncol. 2008 Feb 20;26(6):863-9. |  |
|  |  |  |
| Erlotinib, Paclitaxel | [Phase I, pharmacokinetic, and biological study of erlotinib in combination with paclitaxel and carboplatin in patients with advanced solid tumors.](http://www.ncbi.nlm.nih.gov/pubmed/17189413) | Alopecia rates not reported |
|  | Patnaik A, Wood D, Tolcher AW, Hamilton M, Kreisberg JI, Hammond LA, Schwartz G, Beeram M, Hidalgo M, Mita MM, Wolf J, Nadler P, Rowinsky EK. |  |
|  | Clin Cancer Res. 2006 Dec 15;12(24):7406-13. |  |
|  |  |  |
| Erlotinib, Paclitaxel | [Erlotinib and chemoradiation followed by maintenance erlotinib for locally advanced pancreatic cancer: a phase I study.](http://www.ncbi.nlm.nih.gov/pubmed/16317266) | Use of radiation |
|  | Iannitti D, Dipetrillo T, Akerman P, Barnett JM, Maia-Acuna C, Cruff D, Miner T, Martel D, Cioffi W, Remis M, Kennedy T, Safran H. |  |
|  | Am J Clin Oncol. 2005 Dec;28(6):570-5. |  |
| Erlotinib, Topotecan | None |  |
| Gefitinib, Bleomycin | None |  |
| Gefitinib, Cyclophosphamide | None |  |
| Gefitinib, Dactinomycin | None |  |
| Gefitinib, Doxorubicin | [Phase I trial of liposomal doxorubicin and ZD1839 in patients with refractory gynecological malignancies or metastatic breast cancer.](http://www.ncbi.nlm.nih.gov/pubmed/20405155) | No control group |
|  | Campos SM, Berlin ST, Parker LM, Chen WY, Bunnell CA, Atkinson T, Lee J, Matulonis U, Hirsch MS, Harris L, Krasner CN. |  |
|  | Int J Clin Oncol. 2010 Aug;15(4):390-8. Epub 2010 Apr 20. |  |
|  |  |  |
| Gefitinib, Doxorubicin | [A phase II study of gefitinib for patients with advanced HER-1 expressing synovial sarcoma refractory to doxorubicin-containing regimens.](http://www.ncbi.nlm.nih.gov/pubmed/18448563) | No concurrent therapy |
|  | Ray-Coquard I, Le Cesne A, Whelan JS, Schoffski P, Bui BN, Verweij J, Marreaud S, van Glabbeke M, Hogendoorn P, Blay JY. |  |
|  | Oncologist. 2008 Apr;13(4):467-73. |  |
|  |  |  |
| Gefitinib, Doxorubicin | [Gefitinib (ZD1839) combined with weekly epirubicin in patients with metastatic breast cancer: a phase I study with biological correlate.](http://www.ncbi.nlm.nih.gov/pubmed/16107496) | No control group |
|  | Gasparini G, Sarmiento R, Amici S, Longo R, Gattuso D, Zancan M, Gion M. |  |
|  | Ann Oncol. 2005 Dec;16(12):1867-73. Epub 2005 Aug 17. |  |
| Gefitinib, Irinotecan | [Phase I study of irinotecan and gefitinib in patients with gefitinib treatment failure for non-small cell lung cancer.](http://www.ncbi.nlm.nih.gov/pubmed/21915126) | No control group |
|  | Horiike A, Kudo K, Miyauchi E, Ohyanagi F, Kasahara K, Horai T, Nishio M. |  |
|  | Br J Cancer. 2011 Oct 11;105(8):1131-6. doi: 10.1038/bjc.2011.375. Epub 2011 Sep 13. |  |
|  |  |  |
| Gefitinib, Irinotecan | [A single-arm pilot phase II study of gefitinib and irinotecan in children with newly diagnosed high-risk neuroblastoma.](http://www.ncbi.nlm.nih.gov/pubmed/21796439) | No control group |
|  | Furman WL, McGregor LM, McCarville MB, Onciu M, Davidoff AM, Kovach S, Hawkins D, McPherson V, Houghton PJ, Billups CA, Wu J, Stewart CF, Santana VM. |  |
|  | Invest New Drugs. 2011 Jul 28. [Epub ahead of print] |  |
|  |  |  |
| Gefitinib, Irinotecan | [Phase II study of paclitaxel and irinotecan with intercalated gefitinib in patients with advanced non-small-cell lung cancer.](http://www.ncbi.nlm.nih.gov/pubmed/19786849) | Anti-EGFR administered following chemotherapy |
|  | Oshita F, Saito H, Murakami S, Kondo T, Yamada K. |  |
|  | Am J Clin Oncol. 2010 Feb;33(1):66-9. |  |
|  |  |  |
| Gefitinib, Irinotecan | [A phase II randomized multicenter trial of gefitinib plus FOLFIRI and FOLFIRI alone in patients with metastatic colorectal cancer.](http://www.ncbi.nlm.nih.gov/pubmed/18667394) | Alopecia rates not reported |
|  | Santoro A, Comandone A, Rimassa L, Granetti C, Lorusso V, Oliva C, Ronzoni M, Siena S, Zuradelli M, Mari E, Pressiani T, Carnaghi C. |  |
|  | Ann Oncol. 2008 Nov;19(11):1888-93. Epub 2008 Jul 30. |  |
|  |  |  |
| Gefitinib, Irinotecan | [Gefitinib in combination with oxaliplatin and 5-fluorouracil in irinotecan-refractory patients with colorectal cancer: a phase I study of the Arbeits gemeinschaft Internistische Onkologie (AIO).](http://www.ncbi.nlm.nih.gov/pubmed/18497512) | Results not written in English |
|  | Hartmann JT, Pintoffl JP, Kröning H, Bokemeyer C, Holtmann M, Höhler T. |  |
|  | Onkologie. 2008 May;31(5):237-41. Epub 2008 Apr 10. |  |
|  |  |  |
| Gefitinib, Irinotecan | [Induction chemotherapy with carboplatin, irinotecan, and paclitaxel followed by high dose three-dimension conformal thoracic radiotherapy (74 Gy) with concurrent carboplatin, paclitaxel, and gefitinib in unresectable stage IIIA and stage IIIB non-small cell lung cancer.](http://www.ncbi.nlm.nih.gov/pubmed/18317067) | Use of radiation |
|  | Stinchcombe TE, Morris DE, Lee CB, Moore DT, Hayes DN, Halle JS, Rivera MP, Rosenman JG, Socinski MA. |  |
|  | J Thorac Oncol. 2008 Mar;3(3):250-7. |  |
|  |  |  |
| Gefitinib, Irinotecan | [Phase II study of nedaplatin and irinotecan followed by gefitinib for elderly patients with unresectable non-small cell lung cancer.](http://www.ncbi.nlm.nih.gov/pubmed/17960380) | Anti-EGFR administered following chemotherapy |
|  | Oshita F, Yamada K, Saito H, Noda K. |  |
|  | Cancer Chemother Pharmacol. 2008 Aug;62(3):465-70. Epub 2007 Oct 25. |  |
|  |  |  |
| Gefitinib, Irinotecan | [Gefitinib and irinotecan in patients with fluoropyrimidine-refractory, irinotecan-naive advanced colorectal cancer: a phase I-II study.](http://www.ncbi.nlm.nih.gov/pubmed/17237473) | No control group |
|  | Chau I, Cunningham D, Hickish T, Massey A, Higgins L, Osborne R, Botwood N, Swaisland A. |  |
|  | Ann Oncol. 2007 Apr;18(4):730-7. Epub 2007 Jan 20. |  |
|  |  |  |
| Gefitinib, Irinotecan | [Gefitinib in combination with 5-fluorouracil (5-FU)/folinic acid and irinotecan in patients with 5-FU/oxaliplatin- refractory colorectal cancer: a phase I/II study of the Arbeitsgemeinschaft für Internistische Onkologie (AIO).](http://www.ncbi.nlm.nih.gov/pubmed/17202826) | Results not written in English |
|  | Hofheinz RD, Kubicka S, Wollert J, Arnold D, Hochhaus A. |  |
|  | Onkologie. 2006 Dec;29(12):563-7. Epub 2006 Dec 11. |  |
|  |  |  |
| Gefitinib, Irinotecan | [Phase I study of gefitinib plus FOLFIRI in previously untreated patients with metastatic colorectal cancer.](http://www.ncbi.nlm.nih.gov/pubmed/17026790) | No control group |
|  | Wolpin BM, Clark JW, Meyerhardt JA, Earle CC, Ryan DP, Enzinger PC, Zhu AX, Blaszkowsky L, Battu S, Fuchs CS. |  |
|  | Clin Colorectal Cancer. 2006 Sep;6(3):208-13. |  |
|  |  |  |
| Gefitinib, Irinotecan | [Phase II study of gefitinib, fluorouracil, leucovorin, and oxaliplatin therapy in previously treated patients with metastatic colorectal cancer.](http://www.ncbi.nlm.nih.gov/pubmed/16110021) | No control group |
|  | Kuo T, Cho CD, Halsey J, Wakelee HA, Advani RH, Ford JM, Fisher GA, Sikic BI. |  |
|  | J Clin Oncol. 2005 Aug 20;23(24):5613-9. |  |
|  |  |  |
| Gefitinib, Irinotecan | [A phase II trial of gefitinib with 5-fluorouracil, leucovorin, and irinotecan in patients with colorectal cancer.](http://www.ncbi.nlm.nih.gov/pubmed/15870719) | No control group |
|  | Veronese ML, Sun W, Giantonio B, Berlin J, Shults J, Davis L, Haller DG, O'Dwyer PJ. |  |
|  | Br J Cancer. 2005 May 23;92(10):1846-9. |  |
| Gefitinib, Paclitaxel | [Chemoradiotherapy and gefitinib in stage III non-small cell lung cancer with epidermal growth factor receptor and KRAS mutation analysis: cancer and leukemia group B (CALEB) 30106, a CALGB-stratified phase II trial.](http://www.ncbi.nlm.nih.gov/pubmed/20686428) | Use of radiation |
|  | Ready N, Jänne PA, Bogart J, Dipetrillo T, Garst J, Graziano S, Gu L, Wang X, Green MR, Vokes EE; Cancer, Leukemia Group B, Chicago, IL. |  |
|  | J Thorac Oncol. 2010 Sep;5(9):1382-90. |  |
|  |  |  |
| Gefitinib, Paclitaxel | [Gefitinib or chemotherapy for non-small-cell lung cancer with mutated EGFR.](http://www.ncbi.nlm.nih.gov/pubmed/20573926) | Alopecia rates not reported |
|  | Maemondo M, Inoue A, Kobayashi K, Sugawara S, Oizumi S, Isobe H, Gemma A, Harada M, Yoshizawa H, Kinoshita I, Fujita Y, Okinaga S, Hirano H, Yoshimori K, Harada T, Ogura T, Ando M, Miyazawa H, Tanaka T, Saijo Y, Hagiwara K, Morita S, Nukiwa T; North-East Japan Study Group. |  |
|  | N Engl J Med. 2010 Jun 24;362(25):2380-8. |  |
|  |  |  |
| Gefitinib, Paclitaxel | [Epidermal growth factor receptor inhibitor gefitinib added to chemoradiotherapy in locally advanced head and neck cancer.](http://www.ncbi.nlm.nih.gov/pubmed/20498391) | Use of radiation |
|  | Cohen EE, Haraf DJ, Kunnavakkam R, Stenson KM, Blair EA, Brockstein B, Lester EP, Salama JK, Dekker A, Williams R, Witt ME, Grushko TA, Dignam JJ, Lingen MW, Olopade OI, Vokes EE. |  |
|  | J Clin Oncol. 2010 Jul 10;28(20):3336-43. Epub 2010 May 24. |  |
|  |  |  |
| Gefitinib, Paclitaxel | [Phase II study of gefitinib in combination with paclitaxel (P) and carboplatin (C) as second-line therapy for ovarian, tubal or peritoneal adenocarcinoma (1839IL/0074).](http://www.ncbi.nlm.nih.gov/pubmed/20109725) | No control group |
|  | Pautier P, Joly F, Kerbrat P, Bougnoux P, Fumoleau P, Petit T, Rixe O, Ringeisen F, Carrasco AT, Lhommé C. |  |
|  | Gynecol Oncol. 2010 Feb;116(2):157-62. |  |
|  |  |  |
| Gefitinib, Paclitaxel | [Phase II study of paclitaxel and irinotecan with intercalated gefitinib in patients with advanced non-small-cell lung cancer.](http://www.ncbi.nlm.nih.gov/pubmed/19786849) | Anti-EGFR administered following chemotherapy |
|  | Oshita F, Saito H, Murakami S, Kondo T, Yamada K. |  |
|  | Am J Clin Oncol. 2010 Feb;33(1):66-9. |  |
|  |  |  |
| Gefitinib, Paclitaxel | [Gefitinib plus paclitaxel after failure of gefitinib in non-small cell lung cancer initially responding to gefitinib.](http://www.ncbi.nlm.nih.gov/pubmed/19596955) | No control group |
|  | Shukuya T, Takahashi T, Tamiya A, Ono A, Igawa S, Nakamura Y, Tsuya A, Murakami H, Naito T, Kaira K, Endo M, Yamamoto N. |  |
|  | Anticancer Res. 2009 Jul;29(7):2747-51. |  |
|  |  |  |
| Gefitinib, Paclitaxel | [Induction chemotherapy with carboplatin, irinotecan, and paclitaxel followed by high dose three-dimension conformal thoracic radiotherapy (74 Gy) with concurrent carboplatin, paclitaxel, andgefitinib in unresectable stage IIIA and stage IIIB non-small cell lung cancer.](http://www.ncbi.nlm.nih.gov/pubmed/18317067) | Use of radiation |
|  | Stinchcombe TE, Morris DE, Lee CB, Moore DT, Hayes DN, Halle JS, Rivera MP, Rosenman JG, Socinski MA. |  |
|  | J Thorac Oncol. 2008 Mar;3(3):250-7. |  |
|  |  |  |
| Gefitinib, Paclitaxel | [Paclitaxel and carboplatin as first-line chemotherapy combined with gefitinib (IRESSA) in patients with advanced breast cancer: a phase I/II study conducted by the Hellenic Cooperative Oncology Group.](http://www.ncbi.nlm.nih.gov/pubmed/15980985) | No control group |
|  | Fountzilas G, Pectasides D, Kalogera-Fountzila A, Skarlos D, Kalofonos HP, Papadimitriou C, Bafaloukos D, Lambropoulos S, Papadopoulos S, Kourea H, Markopoulos C, Linardou H, Mavroudis D, Briasoulis E, Pavlidis N, Razis E, Kosmidis P, Gogas H. |  |
|  | Breast Cancer Res Treat. 2005 Jul;92(1):1-9. |  |
|  |  |  |
| Gefitinib, Paclitaxel | [Pilot trial of the epidermal growth factor receptor tyrosine kinase inhibitor gefitinib plus carboplatin and paclitaxel in patients with stage IIIB or IV non-small-cell lung cancer.](http://www.ncbi.nlm.nih.gov/pubmed/12775734) | No control group |
|  | Miller VA, Johnson DH, Krug LM, Pizzo B, Tyson L, Perez W, Krozely P, Sandler A, Carbone D, Heelan RT, Kris MG, Smith R, Ochs J. |  |
|  | J Clin Oncol. 2003 Jun 1;21(11):2094-100. |  |
| Gefitinib, Topotecan | [Gefitinib in combination with oral topotecan and cyclophosphamide in relapsed neuroblastoma: pharmacological rationale and clinical response.](http://www.ncbi.nlm.nih.gov/pubmed/19821523) | No control group |
|  | Donfrancesco A, De Ioris MA, McDowell HP, De Pasquale MD, Ilari I, Jenkner A, Castellano A, Cialfi S, De Laurentis C, Dominici C. |  |
|  | Pediatr Blood Cancer. 2010 Jan;54(1):55-61. |  |
| Cetuximab, Bleomycin | None |  |
| Cetuximab, Cyclophosphamide | None |  |
| Cetuximab, Dactinomycin | None |  |
| Cetuximab, Doxorubicin | [Anti-epidermal growth factor receptor monoclonal antibody cetuximab plus Doxorubicin in the treatment of metastatic castration-resistant prostate cancer.](http://www.ncbi.nlm.nih.gov/pubmed/19815486) | No control group |
|  | Slovin SF, Kelly WK, Wilton A, Kattan M, Myskowski P, Mendelsohn J, Scher HI. |  |
|  | Clin Genitourin Cancer. 2009 Oct;7(3):E77-82. |  |
| Cetuximab, Irinotecan | [Anti-EGFR (cetuximab) combined with irinotecan for treatment of elderly patients with metastatic colorectal cancer (mCRC).](http://www.ncbi.nlm.nih.gov/pubmed/22526166) | No control group |
|  | Abdelwahab S, Azmy A, Abdel-Aziz H, Salim H, Mahmoud A. |  |
|  | J Cancer Res Clin Oncol. 2012 Apr 22. [Epub ahead of print] |  |
|  |  |  |
| Cetuximab, Irinotecan | [Multifactorial pharmacogenetic analysis in colorectal cancer patients receiving 5-fluorouracil-based therapy together with cetuximab-irinotecan.](http://www.ncbi.nlm.nih.gov/pubmed/22486600) | No control group |
|  | Etienne-Grimaldi MC, Bennouna J, Formento JL, Douillard JY, Francoual M, Hennebelle I, Chatelut E, Francois E, Faroux R, El Hannani C, Jacob JH, Milano G. |  |
|  | Br J Clin Pharmacol. 2012 May;73(5):776-785. |  |
|  |  |  |
| Cetuximab, Irinotecan | [Cetuximab Plus Cisplatin, Irinotecan, and Thoracic Radiotherapy as Definitive Treatment for Locally Advanced, Unresectable Esophageal Cancer: A Phase-II Study of The SWOG (S0414).](http://www.ncbi.nlm.nih.gov/pubmed/22481235) | Use of radiation |
|  | Tomblyn MB, Goldman BH, Thomas CR Jr, Benedetti JK, Lenz HJ, Mehta V, Beeker T, Gold PJ, Abbruzzese JL, Blanke CD; for the SWOG GI Committee. |  |
|  | J Thorac Oncol. 2012 Apr 4. |  |
|  |  |  |
| Cetuximab, Irinotecan | [Phase II study of biweekly cetuximab in combination with irinotecan as second-line treatment in patients with platinum-resistant gastro-oesophageal cancer.](http://www.ncbi.nlm.nih.gov/pubmed/22244801) | No control group |
|  | Schønnemann KR, Yilmaz M, Bjerregaard JK, Nielsen KM, Pfeiffer P. |  |
|  | Eur J Cancer. 2012 Mar;48(4):510-7. Epub 2012 Jan 12. |  |
|  |  |  |
| Cetuximab, Irinotecan | [FOLFIRI plus cetuximab versus FOLFIRI plus bevacizumab as first-line treatment for patients with metastatic colorectal cancer-subgroup analysis of patients with KRAS: mutated tumours in the randomised German AIO study KRK-0306.](http://www.ncbi.nlm.nih.gov/pubmed/22219013) | No control group |
|  | Stintzing S, Fischer von Weikersthal L, Decker T, Vehling-Kaiser U, Jäger E, Heintges T, Stoll C, Giessen C, Modest DP, Neumann J, Jung A, Kirchner T, Scheithauer W, Heinemann V. |  |
|  | Ann Oncol. 2012 Jan 4. [Epub ahead of print] |  |
|  |  |  |
| Cetuximab, Irinotecan | [Oxaliplatin, irinotecan and cetuximab in advanced gastric cancer. A multicenter phase II trial (Gastric-2) of the Arbeitsgemeinschaft Medikamentose Tumortherapie (AGMT).](http://www.ncbi.nlm.nih.gov/pubmed/22199312) | No control group |
|  | Wöll E, Greil R, Eisterer W, Bechter O, Fridrik MA, Grünberger B, Zabernigg A, Mayrbäurl B, Russ G, Dlaska M, Obrist P, Thaler J. |  |
|  | Anticancer Res. 2011 Dec;31(12):4439-43. |  |
|  |  |  |
| Cetuximab, Irinotecan | [Phase III Trial of Cetuximab, Bevacizumab, and 5-Fluorouracil/Leucovorin vs. FOLFOX-Bevacizumab in Colorectal Cancer.](http://www.ncbi.nlm.nih.gov/pubmed/22055112) | No control group |
|  | Saltz L, Badarinath S, Dakhil S, Bienvenu B, Harker WG, Birchfield G, Tokaz LK, Barrera D, Conkling PR, O'Rourke MA, Richards DA, Reidy D, Solit D, Vakiani E, Capanu M, Scales A, Zhan F, Boehm KA, Asmar L, Cohn A. |  |
|  | Clin Colorectal Cancer. 2011 Nov 4. [Epub ahead of print] |  |
|  |  |  |
| Cetuximab, Irinotecan | [Cetuximab plus FOLFIRINOX (ERBIRINOX) as first-line treatment for unresectable metastatic colorectal cancer: a phase II trial.](http://www.ncbi.nlm.nih.gov/pubmed/22016477) | No control group |
|  | Assenat E, Desseigne F, Thezenas S, Viret F, Mineur L, Kramar A, Samalin E, Portales F, Bibeau F, Crapez-Lopez E, Bleuse JP, Ychou M. |  |
|  | Oncologist. 2011;16(11):1557-64. Epub 2011 Oct 20. |  |
|  |  |  |
| Cetuximab, Irinotecan | [Phase II study of irinotecan and cetuximab given every 2 weeks as second-line therapy for advanced colorectal cancer.](http://www.ncbi.nlm.nih.gov/pubmed/21813336) | No control group |
|  | Carneiro BA, Ramanathan RK, Fakih MG, Krishnamurthi SS, Lembersky BC, Stoller RG, Lancaster SL, Pinkerton RA, Crandall TL, Schmotzer AR, Potter DM, Bahary N. |  |
|  | Clin Colorectal Cancer. 2012 Mar;11(1):53-9. doi: 10.1016/j.clcc.2011.05.003. Epub 2011 Aug 2. |  |
|  |  |  |
| Cetuximab, Irinotecan | [Biweekly cetuximab plus irinotecan as second-line chemotherapy for patients with irinotecan-refractory and KRAS wild-type metastatic colorectal cancer according to epidermal growth factor receptor expression status.](http://www.ncbi.nlm.nih.gov/pubmed/21706149) | No control group |
|  | Kang MJ, Hong YS, Kim KP, Kim SY, Baek JY, Ryu MH, Lee JL, Chang HM, Kim MJ, Chang HJ, Kang YK, Kim TW. |  |
|  | Invest New Drugs. 2011 Jun 25. [Epub ahead of print] |  |
|  |  |  |
| Cetuximab, Irinotecan | [Cetuximab plus irinotecan, fluorouracil, and leucovorin as first-line treatment for metastatic colorectal cancer: updated analysis of overall survival according to tumor KRAS and BRAF mutation status.](http://www.ncbi.nlm.nih.gov/pubmed/21502544) | Alopecia rates not reported |
|  | Van Cutsem E, Köhne CH, Láng I, Folprecht G, Nowacki MP, Cascinu S, Shchepotin I, Maurel J, Cunningham D, Tejpar S, Schlichting M, Zubel A, Celik I, Rougier P, Ciardiello F. |  |
|  | J Clin Oncol. 2011 May 20;29(15):2011-9. Epub 2011 Apr 18. |  |
|  |  |  |
| Cetuximab, Irinotecan | [Biweekly cetuximab and irinotecan as second-line therapy in patients with gastro-esophageal cancer previously treated with platinum.](http://www.ncbi.nlm.nih.gov/pubmed/21409520) | No control group |
|  | Schoennemann KR, Bjerregaard JK, Hansen TP, De Stricker K, Gjerstorff MF, Jensen HA, Vestermark LW, Pfeiffer P. |  |
|  | Gastric Cancer. 2011 Aug;14(3):219-25. doi: 10.1007/s10120-011-0031-7. Epub 2011 Mar 17. |  |
|  |  |  |
| Cetuximab, Irinotecan | [Cetuximab plus capecitabine and irinotecan compared with cetuximab plus capecitabine and oxaliplatin as first-line treatment for patients with metastatic colorectal cancer: AIO KRK-0104--a randomized trial of the German AIO CRC study group.](http://www.ncbi.nlm.nih.gov/pubmed/21300933) | No control group |
|  | Moosmann N, von Weikersthal LF, Vehling-Kaiser U, Stauch M, Hass HG, Dietzfelbinger H, Oruzio D, Klein S, Zellmann K, Decker T, Schulze M, Abenhardt W, Puchtler G, Kappauf H, Mittermüller J, Haberl C, Schalhorn A, Jung A, Stintzing S, Heinemann V. |  |
|  | J Clin Oncol. 2011 Mar 10;29(8):1050-8. Epub 2011 Feb 7. |  |
|  |  |  |
| Cetuximab, Irinotecan | [Cetuximab Plus irinotecan in pretreated metastatic colorectal cancer progressing on irinotecan: the LABEL study.](http://www.ncbi.nlm.nih.gov/pubmed/21208842) | No control group |
|  | Buzaid AC, Mathias Cde C, Perazzo F, Simon SD, Fein L, Hidalgo J, Murad AM, Esser R, Senger S, Lerzo G. |  |
|  | Clin Colorectal Cancer. 2010 Dec;9(5):282-9. |  |
|  |  |  |
| Cetuximab, Irinotecan | [Phase II study of combination chemotherapy with biweekly cetuximab and irinotecan for wild-type KRAS metastatic colorectal cancer refractory to irinotecan, oxaliplatin, and fluoropyrimidines.](http://www.ncbi.nlm.nih.gov/pubmed/21174225) | No control group |
|  | Shitara K, Yuki S, Yoshida M, Takahari D, Utsunomiya S, Yokota T, Sato Y, Inaba Y, Tajika M, Kawai H, Yamaura H, Kato M, Yamazaki K, Komatsu Y, Muro K. |  |
|  | Invest New Drugs. 2012 Apr;30(2):787-93. Epub 2010 Dec 22. |  |
|  |  |  |
| Cetuximab, Irinotecan | [Cetuximab with irinotecan, folinic acid and 5-fluorouracil as first-line treatment in advanced gastroesophageal cancer: a prospective multi-center biomarker-oriented phase II study.](http://www.ncbi.nlm.nih.gov/pubmed/21119032) | No control group |
|  | Moehler M, Mueller A, Trarbach T, Lordick F, Seufferlein T, Kubicka S, Geissler M, Schwarz S, Galle PR, Kanzler S; German Arbeitsgemeinschaft Internistische Onkologie. |  |
|  | Ann Oncol. 2011 Jun;22(6):1358-66. Epub 2010 Nov 30. |  |
|  |  |  |
| Cetuximab, Irinotecan | [Cetuximab plus chronomodulated irinotecan, 5-fluorouracil, leucovorin and oxaliplatin as neoadjuvant chemotherapy in colorectal liver metastases: POCHER trial.](http://www.ncbi.nlm.nih.gov/pubmed/20959822) | No control group |
|  | Garufi C, Torsello A, Tumolo S, Ettorre GM, Zeuli M, Campanella C, Vennarecci G, Mottolese M, Sperduti I, Cognetti F. |  |
|  | Br J Cancer. 2010 Nov 9;103(10):1542-7. Epub 2010 Oct 19. |  |
|  |  |  |
| Cetuximab, Irinotecan | [Preoperative chemoradiation with cetuximab, irinotecan, and capecitabine in patients with locally advanced resectable rectal cancer: a multicenter Phase II study.](http://www.ncbi.nlm.nih.gov/pubmed/20888703) | Use of radiation |
|  | Kim SY, Hong YS, Kim DY, Kim TW, Kim JH, Im SA, Lee KS, Yun T, Jeong SY, Choi HS, Lim SB, Chang HJ, Jung KH. |  |
|  | Int J Radiat Oncol Biol Phys. 2011 Nov 1;81(3):677-83. Epub 2010 Oct 1. |  |
|  |  |  |
| Cetuximab, Irinotecan | [Cetuximab plus FOLFOX6 or FOLFIRI in metastatic colorectal cancer: CECOG trial.](http://www.ncbi.nlm.nih.gov/pubmed/20593498) | Alopecia rates not reported |
|  | Ocvirk J, Brodowicz T, Wrba F, Ciuleanu TE, Kurteva G, Beslija S, Koza I, Pápai Z, Messinger D, Yilmaz U, Faluhelyi Z, Yalcin S, Papamichael D, Wenczl M, Mrsic-Krmpotic Z, Shacham-Shmueli E, Vrbanec D, Esser R, Scheithauer W, Zielinski CC. |  |
|  | World J Gastroenterol. 2010 Jul 7;16(25):3133-43. |  |
|  |  |  |
| Cetuximab, Irinotecan | [Cetuximab given every 2 weeks plus irinotecan is an active and safe option for previously treated patients with metastatic colorectal cancer.](http://www.ncbi.nlm.nih.gov/pubmed/20407241) | No control group |
|  | Roca JM, Alonso V, Pericay C, Escudero P, Salud A, Losa F, López LJ, Guasch I, Méndez M, Quintero-Aldana G, Grande C, Vicente P, Arrivi A, Martin C, Moreno I, García P, Antón I, Constenla M, Yubero A, Cirera L; ACROSS Cooperative Group. |  |
|  | Chemotherapy. 2010;56(2):142-6. Epub 2010 Apr 21. |  |
|  |  |  |
| Cetuximab, Irinotecan | [Phase II study of combination chemotherapy with irinotecan and cetuximab for pretreated metastatic colorectal cancer harboring wild-type KRAS.](http://www.ncbi.nlm.nih.gov/pubmed/20072801) | No control group |
|  | Shitara K, Yokota T, Takahari D, Shibata T, Ura T, Utsunomiya S, Inaba Y, Yamaura H, Sato Y, Najima M, Kawai H, Tajika M, Sawaki A, Yatabe Y, Muro K. |  |
|  | Invest New Drugs. 2011 Aug;29(4):688-93. Epub 2010 Jan 14. |  |
|  |  |  |
| Cetuximab, Irinotecan | [Tumour response and secondary resectability of colorectal liver metastases following neoadjuvant chemotherapy with cetuximab: the CELIM randomised phase 2 trial.](http://www.ncbi.nlm.nih.gov/pubmed/19942479) | No control group |
|  | Folprecht G, Gruenberger T, Bechstein WO, Raab HR, Lordick F, Hartmann JT, Lang H, Frilling A, Stoehlmacher J, Weitz J, Konopke R, Stroszczynski C, Liersch T, Ockert D, Herrmann T, Goekkurt E, Parisi F, Köhne CH. |  |
|  | Lancet Oncol. 2010 Jan;11(1):38-47. Epub 2009 Nov 26. |  |
|  |  |  |
| Cetuximab, Irinotecan | [Phase I and pharmacokinetic study of cetuximab and irinotecan in children with refractory solid tumors: a study of the pediatric oncology experimental therapeutic investigators' consortium.](http://www.ncbi.nlm.nih.gov/pubmed/19770383) | No control group |
|  | Trippett TM, Herzog C, Whitlock JA, Wolff J, Kuttesch J, Bagatell R, Hunger SP, Boklan J, Smith AA, Arceci RJ, Katzenstein HM, Harbison C, Zhou X, Lu H, Langer C, Weber M, Gore L. |  |
|  | J Clin Oncol. 2009 Oct 20;27(30):5102-8. Epub 2009 Sep 21. |  |
|  |  |  |
| Cetuximab, Irinotecan | [[Cetuximab and irinotecan for the treatment of metastatic colorectal cancer--pilot results from Masaryk Memorial Cancer Institute].](http://www.ncbi.nlm.nih.gov/pubmed/19534437) | Results not written in English |
|  | Nēmecek R, Kocáková I, Kocák I, Svoboda M, Lakomý R, Poprach A, Vyskocil J, Vyzula R. |  |
|  | Klin Onkol. 2009;22(1):27-33. Czech. |  |
|  |  |  |
| Cetuximab, Irinotecan | [Cetuximab in combination with capecitabine, irinotecan, and radiotherapy for patients with locally advanced rectal cancer: results of a Phase II MARGIT trial.](http://www.ncbi.nlm.nih.gov/pubmed/19131187) | No control group |
|  | Horisberger K, Treschl A, Mai S, Barreto-Miranda M, Kienle P, Ströbel P, Erben P, Woernle C, Dinter D, Kähler G, Hochhaus A, Post S, Willeke F, Wenz F, Hofheinz RD; MARGIT (Mannheimer Arbeitsgruppe für Gastrointestinale Tumoren). |  |
|  | Int J Radiat Oncol Biol Phys. 2009 Aug 1;74(5):1487-93. Epub 2009 Jan 7. |  |
|  |  |  |
| Cetuximab, Irinotecan | [Results of a phase II trial of cetuximab plus capecitabine/irinotecan as first-line therapy for patients with advanced and/or metastatic colorectal cancer.](http://www.ncbi.nlm.nih.gov/pubmed/19036692) | No control group |
|  | Cartwright T, Kuefler P, Cohn A, Hyman W, Berger M, Richards D, Vukelja S, Nugent JE, Ruxer RL Jr, Boehm KA, Asmar L. |  |
|  | Clin Colorectal Cancer. 2008 Nov;7(6):390-7. |  |
|  |  |  |
| Cetuximab, Irinotecan | [Cetuximab plus irinotecan in heavily pretreated metastatic colorectal cancer progressing on irinotecan: MABEL Study.](http://www.ncbi.nlm.nih.gov/pubmed/18854570) | No control group |
|  | Wilke H, Glynne-Jones R, Thaler J, Adenis A, Preusser P, Aguilar EA, Aapro MS, Esser R, Loos AH, Siena S. |  |
|  | J Clin Oncol. 2008 Nov 20;26(33):5335-43. Epub 2008 Oct 14 |  |
|  |  |  |
| Cetuximab, Irinotecan | [Multicenter Phase II study of cetuximab plus irinotecan in metastatic colorectal carcinoma refractory toirinotecan, oxaliplatin and fluoropyrimidines.](http://www.ncbi.nlm.nih.gov/pubmed/18836202) | No control group |
|  | Tahara M, Shirao K, Boku N, Yamaguchi K, Komatsu Y, Inaba Y, Arai T, Mizunuma N, Satoh T, Takiuchi H, Nishina T, Sakata Y. |  |
|  | Jpn J Clin Oncol. 2008 Nov;38(11):762-9. Epub 2008 Oct 4. |  |
|  |  |  |
| Cetuximab, Irinotecan | [Biweekly cetuximab and irinotecan in advanced colorectal cancer patients progressing after at least one previous line of chemotherapy: results of a phase II single institution trial.](http://www.ncbi.nlm.nih.gov/pubmed/18665167) | No control group |
|  | Martín-Martorell P, Roselló S, Rodríguez-Braun E, Chirivella I, Bosch A, Cervantes A. |  |
|  | Br J Cancer. 2008 Aug 5;99(3):455-8. |  |
|  |  |  |
| Cetuximab, Irinotecan | [A phase II study of cetuximab/irinotecan in patients with heavily pretreated metastatic colorectal cancer: predictive value of early specific toxicities.](http://www.ncbi.nlm.nih.gov/pubmed/18650196) | No control group |
|  | Gamucci T, Nelli F, Cianci G, Grassi G, Moscetti L, Sperduti I, Zeuli M, Cortesi E, D'Auria G, Pollera CF. |  |
|  | Clin Colorectal Cancer. 2008 Jul;7(4):273-9. |  |
|  |  |  |
| Cetuximab, Irinotecan | [Cetuximab in combination with weekly 5-fluorouracil/folinic acid and oxaliplatin (FUFOX) in untreated patients with advanced colorectal cancer: a phase Ib/II study of the AIO GI Group.](http://www.ncbi.nlm.nih.gov/pubmed/18441330) | No control group |
|  | Arnold D, Höhler T, Dittrich C, Lordick F, Seufferlein T, Riemann J, Wöll E, Herrmann T, Zubel A, Schmoll HJ. |  |
|  | Ann Oncol. 2008 Aug;19(8):1442-9. Epub 2008 Apr 25. |  |
|  |  |  |
| Cetuximab, Irinotecan | [EPIC: phase III trial of cetuximab plus irinotecan after fluoropyrimidine and oxaliplatin failure in patients with metastatic colorectal cancer.](http://www.ncbi.nlm.nih.gov/pubmed/18390971) | No concurrent anti-EGFR therapy |
|  | Sobrero AF, Maurel J, Fehrenbacher L, Scheithauer W, Abubakr YA, Lutz MP, Vega-Villegas ME, Eng C, Steinhauer EU, Prausova J, Lenz HJ, Borg C, Middleton G, Kröning H, Luppi G, Kisker O, Zubel A, Langer C, Kopit J, Burris HA 3rd. |  |
|  | J Clin Oncol. 2008 May 10;26(14):2311-9. Epub 2008 Apr 7. |  |
|  |  |  |
| Cetuximab, Irinotecan | [Biweekly cetuximab and irinotecan as third-line therapy in patients with advanced colorectal cancer after failure to irinotecan, oxaliplatin and 5-fluorouracil.](http://www.ncbi.nlm.nih.gov/pubmed/18281264) | No control group |
|  | Pfeiffer P, Nielsen D, Bjerregaard J, Qvortrup C, Yilmaz M, Jensen B. |  |
|  | Ann Oncol. 2008 Jun;19(6):1141-5. Epub 2008 Feb 14. |  |
|  |  |  |
| Cetuximab, Irinotecan | [A Phase II study of cetuximab (Erbitux) plus FOLFIRI for irinotecan and oxaliplatin-refractory metastatic colorectal cancer.](http://www.ncbi.nlm.nih.gov/pubmed/17923763) | No control group |
|  | Koo DH, Lee JL, Kim TW, Chang HM, Ryu MH, Lee SS, Kim MK, Sym SJ, Lee JS, Kang YK. |  |
|  | J Korean Med Sci. 2007 Sep;22 Suppl:S98-S103. |  |
|  |  |  |
| Cetuximab, Irinotecan | [Randomized phase II trial of cetuximab, bevacizumab, and irinotecan compared with cetuximab and bevacizumab alone in irinotecan-refractory colorectal cancer: the BOND-2 study.](http://www.ncbi.nlm.nih.gov/pubmed/17876013) | No concurrent chemotherapy |
|  | Saltz LB, Lenz HJ, Kindler HL, Hochster HS, Wadler S, Hoff PM, Kemeny NE, Hollywood EM, Gonen M, Quinones M, Morse M, Chen HX. |  |
|  | J Clin Oncol. 2007 Oct 10;25(29):4557-61. Epub 2007 Sep 17. |  |
|  |  |  |
| Cetuximab, Irinotecan | [Cetuximab and irinotecan as third line therapy in patients with advanced colorectal cancer after failure ofirinotecan, oxaliplatin and 5-fluorouracil.](http://www.ncbi.nlm.nih.gov/pubmed/17562448) | No control group |
|  | Pfeiffer P, Nielsen D, Yilmaz M, Iversen A, Vejlø C, Jensen BV. |  |
|  | Acta Oncol. 2007;46(5):697-701. |  |
|  |  |  |
| Cetuximab, Irinotecan | [Phase II trial of capecitabine and oxaliplatin (CAPOX) plus cetuximab in patients with metastatic colorectal cancer who progressed after oxaliplatin-based chemotherapy.](http://www.ncbi.nlm.nih.gov/pubmed/17079693) | No control group |
|  | Souglakos J, Kalykaki A, Vamvakas L, Androulakis N, Kalbakis K, Agelaki S, Vardakis N, Tzardi M, Kotsakis AP, Gioulbasanis J, Tsetis D, Sfakiotaki G, Chatzidaki D, Mavroudis D, Georgoulias V. |  |
|  | Ann Oncol. 2007 Feb;18(2):305-10. Epub 2006 Nov 1. |  |
|  |  |  |
| Cetuximab, Irinotecan | [Multicenter phase II and translational study of cetuximab in metastatic colorectal carcinoma refractory toirinotecan, oxaliplatin, and fluoropyrimidines.](http://www.ncbi.nlm.nih.gov/pubmed/17050875) | No control group |
|  | Lenz HJ, Van Cutsem E, Khambata-Ford S, Mayer RJ, Gold P, Stella P, Mirtsching B, Cohn AL, Pippas AW, Azarnia N, Tsuchihashi Z, Mauro DJ, Rowinsky EK. |  |
|  | J Clin Oncol. 2006 Oct 20;24(30):4914-21. |  |
|  |  |  |
| Cetuximab, Irinotecan | [Phase I trial of cetuximab in combination with capecitabine, weekly irinotecan, and radiotherapy as neoadjuvant therapy for rectal cancer.](http://www.ncbi.nlm.nih.gov/pubmed/16979839) | Use of radiation |
|  | Hofheinz RD, Horisberger K, Woernle C, Wenz F, Kraus-Tiefenbacher U, Kähler G, Dinter D, Grobholz R, Heeger S, Post S, Hochhaus A, Willeke F. |  |
|  | Int J Radiat Oncol Biol Phys. 2006 Dec 1;66(5):1384-90. Epub 2006 Sep 18. |  |
|  |  |  |
| Cetuximab, Irinotecan | [Cetuximab and irinotecan as third-line therapy in advanced colorectal cancer patients: a single centre phase II trial.](http://www.ncbi.nlm.nih.gov/pubmed/16508634) | No control group |
|  | Vincenzi B, Santini D, Rabitti C, Coppola R, Beomonte Zobel B, Trodella L, Tonini G. |  |
|  | Br J Cancer. 2006 Mar 27;94(6):792-7. |  |
|  |  |  |
| Cetuximab, Irinotecan | [Cetuximab and irinotecan/5-fluorouracil/folinic acid is a safe combination for the first-line treatment of patients with epidermal growth factor receptor expressing metastatic colorectal carcinoma.](http://www.ncbi.nlm.nih.gov/pubmed/16303861) | No control group |
|  | Folprecht G, Lutz MP, Schöffski P, Seufferlein T, Nolting A, Pollert P, Köhne CH. |  |
|  | Ann Oncol. 2006 Mar;17(3):450-6. Epub 2005 Nov 22. |  |
|  |  |  |
| Cetuximab, Irinotecan | [Pharmacokinetic profile of cetuximab (Erbitux) alone and in combination with irinotecan in patients with advanced EGFR-positive adenocarcinoma.](http://www.ncbi.nlm.nih.gov/pubmed/16051481) | No control group |
|  | Delbaldo C, Pierga JY, Dieras V, Faivre S, Laurence V, Vedovato JC, Bonnay M, Mueser M, Nolting A, Kovar A, Raymond E. |  |
|  | Eur J Cancer. 2005 Aug;41(12):1739-45. |  |
|  |  |  |
| Cetuximab, Irinotecan | [Cetuximab monotherapy and cetuximab plus irinotecan in irinotecan-refractory metastatic colorectal cancer.](http://www.ncbi.nlm.nih.gov/pubmed/15269313) | No control group |
|  | Cunningham D, Humblet Y, Siena S, Khayat D, Bleiberg H, Santoro A, Bets D, Mueser M, Harstrick A, Verslype C, Chau I, Van Cutsem E. |  |
|  | N Engl J Med. 2004 Jul 22;351(4):337-45. |  |
|  |  |  |
| Cetuximab, Irinotecan | [Phase II trial of cetuximab in patients with refractory colorectal cancer that expresses the epidermal growth factor receptor.](http://www.ncbi.nlm.nih.gov/pubmed/14993230) | No concurrent chemotherapy |
|  | Saltz LB, Meropol NJ, Loehrer PJ Sr, Needle MN, Kopit J, Mayer RJ. |  |
|  | J Clin Oncol. 2004 Apr 1;22(7):1201-8. Epub 2004 Mar 1. |  |
| Cetuximab, Paclitaxel | [Phase II study of the combination of cetuximab and weekly paclitaxel in the first-line treatment of patients with recurrent and/or metastatic squamous cell carcinoma of head and neck.](http://www.ncbi.nlm.nih.gov/pubmed/21865152) | No control group |
|  | Hitt R, Irigoyen A, Cortes-Funes H, Grau JJ, García-Sáenz JA, Cruz-Hernandez JJ; Spanish Head and Neck Cancer Cooperative Group (TTCC). |  |
|  | Ann Oncol. 2012 Apr;23(4):1016-22. Epub 2011 Aug 23. |  |
|  |  |  |
| Cetuximab, Paclitaxel | [Phase II study evaluating the addition of cetuximab to the concurrent delivery of weekly carboplatin,paclitaxel, and daily radiotherapy for patients with locally advanced squamous cell carcinomas of the head and neck.](http://www.ncbi.nlm.nih.gov/pubmed/21601372) | Use of radiation |
|  | Suntharalingam M, Kwok Y, Goloubeva O, Parekh A, Taylor R, Wolf J, Zimrin A, Strome S, Ord R, Cullen KJ. |  |
|  | Int J Radiat Oncol Biol Phys. 2012 Apr 1;82(5):1845-50. Epub 2011 May 19. |  |
|  |  |  |
| Cetuximab, Paclitaxel | [Phase II study of cetuximab in combination with chemoradiation in patients with stage IIIA/B non-small-cell lung cancer: RTOG 0324.](http://www.ncbi.nlm.nih.gov/pubmed/21555682) | No control group |
|  | Blumenschein GR Jr, Paulus R, Curran WJ, Robert F, Fossella F, Werner-Wasik M, Herbst RS, Doescher PO, Choy H, Komaki R. |  |
|  | J Clin Oncol. 2011 Jun 10;29(17):2312-8. Epub 2011 May 9. |  |
|  |  |  |
| Cetuximab, Paclitaxel | [Phase II selection design trial of concurrent chemotherapy and cetuximab versus chemotherapy followed by cetuximab in advanced-stage non-small-cell lung cancer: Southwest Oncology Group study S0342.](http://www.ncbi.nlm.nih.gov/pubmed/20921467) | No control group |
|  | Herbst RS, Kelly K, Chansky K, Mack PC, Franklin WA, Hirsch FR, Atkins JN, Dakhil SR, Albain KS, Kim ES, Redman M, Crowley JJ, Gandara DR. |  |
|  | J Clin Oncol. 2010 Nov 1;28(31):4747-54. Epub 2010 Oct 4. |  |
|  |  |  |
| Cetuximab, Paclitaxel | [Cetuximab and first-line taxane/carboplatin chemotherapy in advanced non-small-cell lung cancer: results of the randomized multicenter phase III trial BMS099.](http://www.ncbi.nlm.nih.gov/pubmed/20100966) | Alopecia rates not reported |
|  | Lynch TJ, Patel T, Dreisbach L, McCleod M, Heim WJ, Hermann RC, Paschold E, Iannotti NO, Dakhil S, Gorton S, Pautret V, Weber MR, Woytowitz D. |  |
|  | J Clin Oncol. 2010 Feb 20;28(6):911-7. Epub 2010 Jan 25. |  |
|  |  |  |
| Cetuximab, Paclitaxel | [Induction chemotherapy and cetuximab for locally advanced squamous cell carcinoma of the head and neck: results from a phase II prospective trial.](http://www.ncbi.nlm.nih.gov/pubmed/19917840) | No control group |
|  | Kies MS, Holsinger FC, Lee JJ, William WN Jr, Glisson BS, Lin HY, Lewin JS, Ginsberg LE, Gillaspy KA, Massarelli E, Byers L, Lippman SM, Hong WK, El-Naggar AK, Garden AS, Papadimitrakopoulou V. |  |
|  | J Clin Oncol. 2010 Jan 1;28(1):8-14. Epub 2009 Nov 16. |  |
|  |  |  |
| Cetuximab, Paclitaxel | [A randomized, phase II trial of two dose schedules of carboplatin/paclitaxel/cetuximab in stage IIIB/IV non-small-cell lung cancer (NSCLC).](http://www.ncbi.nlm.nih.gov/pubmed/19188136) | Alopecia rates not reported |
|  | Socinski MA, Saleh MN, Trent DF, Dobbs TW, Zehngebot LM, Levine MA, Bordoni R, Stella PJ. |  |
|  | Ann Oncol. 2009 Jun;20(6):1068-73. Epub 2009 Feb 2. |  |
|  |  |  |
| Cetuximab, Paclitaxel | [Phase II study of paclitaxel, carboplatin, and cetuximab as first line treatment, for patients with advanced non-small cell lung cancer (NSCLC): results of OPN-017.](http://www.ncbi.nlm.nih.gov/pubmed/18978564) | No control group |
|  | Borghaei H, Langer CJ, Millenson M, Ruth KJ, Litwin S, Tuttle H, Seldomridge JS, Rovito M, Mintzer D, Cohen R, Treat J. |  |
|  | J Thorac Oncol. 2008 Nov;3(11):1286-92. |  |
|  |  |  |
| Cetuximab, Paclitaxel | [A phase II study of cetuximab/paclitaxel/carboplatin for the initial treatment of advanced-stage ovarian, primary peritoneal, or fallopian tube cancer.](http://www.ncbi.nlm.nih.gov/pubmed/18554700) | No control group |
|  | Konner J, Schilder RJ, DeRosa FA, Gerst SR, Tew WP, Sabbatini PJ, Hensley ML, Spriggs DR, Aghajanian CA. |  |
|  | Gynecol Oncol. 2008 Aug;110(2):140-5. Epub 2008 Jun 13. |  |
|  |  |  |
| Cetuximab, Paclitaxel | [A phase I study of cetuximab/paclitaxel in patients with advanced-stage breast cancer.](http://www.ncbi.nlm.nih.gov/pubmed/16942645) | No control group |
|  | Modi S, D'Andrea G, Norton L, Yao TJ, Caravelli J, Rosen PP, Hudis C, Seidman AD. |  |
|  | Clin Breast Cancer. 2006 Aug;7(3):270-7. |  |
|  |  |  |
| Cetuximab, Paclitaxel | [Multicenter phase I/II study of cetuximab with paclitaxel and carboplatin in untreated patients with stage IV non-small-cell lung cancer.](http://www.ncbi.nlm.nih.gov/pubmed/16246975) | No control group |
|  | Thienelt CD, Bunn PA Jr, Hanna N, Rosenberg A, Needle MN, Long ME, Gustafson DL, Kelly K. |  |
|  | J Clin Oncol. 2005 Dec 1;23(34):8786-93. Epub 2005 Oct 24. |  |
|  |  |  |
| Cetuximab, Paclitaxel | [Phase II multicenter study of the epidermal growth factor receptor antibody cetuximab and cisplatin for recurrent and refractory squamous cell carcinoma of the head and neck.](http://www.ncbi.nlm.nih.gov/pubmed/16009949) | Anti-EGFR administered following chemotherapy |
|  | Herbst RS, Arquette M, Shin DM, Dicke K, Vokes EE, Azarnia N, Hong WK, Kies MS. |  |
|  | J Clin Oncol. 2005 Aug 20;23(24):5578-87. Epub 2005 Jul 11. |  |
| Cetuximab, Topotecan | [Cetuximab, topotecan and cisplatin for the treatment of advanced cervical cancer: A phase II GINECO trial.](http://www.ncbi.nlm.nih.gov/pubmed/19232434) | No control group |
|  | Kurtz JE, Hardy-Bessard AC, Deslandres M, Lavau-Denes S, Largillier R, Roemer-Becuwe C, Weber B, Guillemet C, Paraiso D, Pujade-Lauraine E. |  |
|  | Gynecol Oncol. 2009 Apr;113(1):16-20. Epub 2009 Feb 15. |  |
| Panitumumab, Bleomycin | None |  |
| Panitumumab, Cyclophosphamide | None |  |
| Panitumumab, Dactinomycin | None |  |
| Panitumumab, Doxorubicin | None |  |
| Panitumumab, Irinotecan | [The efficacy and safety of panitumumab administered concomitantly with FOLFIRI or Irinotecan in second-line therapy for metastatic colorectal cancer: the secondary analysis from STEPP (Skin Toxicity Evaluation Protocol With Panitumumab) by KRAS status.](http://www.ncbi.nlm.nih.gov.cuhsl.creighton.edu/pubmed/22000810) | No control group |
|  | Mitchell EP, Piperdi B, Lacouture ME, Shearer H, Iannotti N, Pillai MV, Xu F, Yassine M. |  |
|  | Clin Colorectal Cancer. 2011 Dec;10(4):333-9. Epub 2011 Oct 14. |  |
|  |  |  |
| Panitumumab, Irinotecan | [First-line panitumumab plus irinotecan/5-fluorouracil/leucovorin treatment in patients with metastatic colorectal cancer.](http://www.ncbi.nlm.nih.gov.cuhsl.creighton.edu/pubmed/21960318) | No control group |
|  | Köhne CH, Hofheinz R, Mineur L, Letocha H, Greil R, Thaler J, Fernebro E, Gamelin E, Decosta L, Karthaus M. |  |
|  | J Cancer Res Clin Oncol. 2012 Jan;138(1):65-72. Epub 2011 Sep 30. |  |
|  |  |  |
| Panitumumab, Irinotecan | [Panitumumab in combination with cytotoxic chemotherapy for the treatment of metastatic colorectal carcinoma.](http://www.ncbi.nlm.nih.gov.cuhsl.creighton.edu/pubmed/21925954) | No control group |
|  | Peeters M, Cohn A, Köhne CH, Douillard JY. |  |
|  | Clin Colorectal Cancer. 2012 Mar;11(1):14-23. doi: 10.1016/j.clcc.2011.06.010. Epub 2011 Sep 16. |  |
|  |  |  |
| Panitumumab, Irinotecan | [An open-label, single-arm, phase 2 trial of panitumumab plus FOLFIRI as second-line therapy in patients with metastatic colorectal cancer.](http://www.ncbi.nlm.nih.gov.cuhsl.creighton.edu/pubmed/21855038) | No control group |
|  | Cohn AL, Shumaker GC, Khandelwal P, Smith DA, Neubauer MA, Mehta N, Richards D, Watkins DL, Zhang K, Yassine MR. |  |
|  | Clin Colorectal Cancer. 2011 Sep;10(3):171-7. doi: 10.1016/j.clcc.2011.03.022. Epub 2011 Apr 28. |  |
|  |  |  |
| Panitumumab, Irinotecan | [Randomized phase III study of panitumumab with fluorouracil, leucovorin, and irinotecan(FOLFIRI) compared with FOLFIRI alone as second-line treatment in patients with metastatic colorectal cancer.](http://www.ncbi.nlm.nih.gov.cuhsl.creighton.edu/pubmed/20921462) | Alopecia rates not reported |
|  | Peeters M, Price TJ, Cervantes A, Sobrero AF, Ducreux M, Hotko Y, André T, Chan E, Lordick F, Punt CJ, Strickland AH, Wilson G, Ciuleanu TE, Roman L, Van Cutsem E, Tzekova V, Collins S, Oliner KS, Rong A, Gansert J. |  |
|  | J Clin Oncol. 2010 Nov 1;28(31):4706-13. Epub 2010 Oct 4. |  |
|  |  |  |
| Panitumumab, Irinotecan | [A phase 2 clinical trial of panitumumab monotherapy in Japanese patients with metastatic colorectal cancer.](http://www.ncbi.nlm.nih.gov.cuhsl.creighton.edu/pubmed/19287023) | No concurrent chemotherapy |
|  | Muro K, Yoshino T, Doi T, Shirao K, Takiuchi H, Hamamoto Y, Watanabe H, Yang BB, Asahi D. |  |
|  | Jpn J Clin Oncol. 2009 May;39(5):321-6. Epub 2009 Mar 14. |  |
|  |  |  |
| Panitumumab, Irinotecan | [Panitumumab monotherapy in patients with previously treated metastatic colorectal cancer.](http://www.ncbi.nlm.nih.gov.cuhsl.creighton.edu/pubmed/17671985) | No concurrent chemotherapy |
|  | Hecht JR, Patnaik A, Berlin J, Venook A, Malik I, Tchekmedyian S, Navale L, Amado RG, Meropol NJ. |  |
|  | Cancer. 2007 Sep 1;110(5):980-8. |  |
|  |  |  |
| Panitumumab, Irinotecan | [Panitumumab with irinotecan/leucovorin/5-fluorouracil for first-line treatment of metastatic colorectal cancer.](http://www.ncbi.nlm.nih.gov.cuhsl.creighton.edu/pubmed/17531105) | No control group |
|  | Berlin J, Posey J, Tchekmedyian S, Hu E, Chan D, Malik I, Yang L, Amado RG, Hecht JR. |  |
|  | Clin Colorectal Cancer. 2007 Mar;6(6):427-32. |  |
| Panitumumab, Paclitaxel | [Phase 1b study of motesanib, an oral angiogenesis inhibitor, in combination with carboplatin/paclitaxel and/or panitumumab for the treatment of advanced non-small cell lung cancer.](http://www.ncbi.nlm.nih.gov.cuhsl.creighton.edu/pubmed/20028752) | Alopecia rates not reported |
|  | Blumenschein GR Jr, Reckamp K, Stephenson GJ, O'Rourke T, Gladish G, McGreivy J, Sun YN, Ye Y, Parson M, Sandler A. |  |
|  | Clin Cancer Res. 2010 Jan 1;16(1):279-90. Epub 2009 Dec 22. |  |
|  |  |  |
| Panitumumab, Paclitaxel | [Phase I dose-finding study of paclitaxel with panitumumab, carboplatin and intensity-modulated radiotherapy in patients with locally advanced squamous cell cancer of the head and neck.](http://www.ncbi.nlm.nih.gov.cuhsl.creighton.edu/pubmed/19892746) | Use of radiation |
|  | Wirth LJ, Allen AM, Posner MR, Haddad RI, Li Y, Clark JR, Busse PM, Chan AW, Goguen LA, Norris CM, Annino DJ, Tishler RB. |  |
|  | Ann Oncol. 2010 Feb;21(2):342-7. Epub 2009 Nov 5. |  |
| Panitumumab, Topotecan | None |  |
